# Lipid Binding of the Amphipathic Helix Serving as Membrane Anchor of Pestivirus Glycoprotein E^rns^

**DOI:** 10.1371/journal.pone.0135680

**Published:** 2015-08-13

**Authors:** Daniel Aberle, Kay-Marcus Oetter, Gregor Meyers

**Affiliations:** Institut für Immunologie, Friedrich-Loeffler-Institut, Greifswald—Insel Riems, Germany; Institut Jacque Monod, Centre National de la Recherche Scientifique, FRANCE

## Abstract

Pestiviruses express a peculiar protein named E^rns^ representing envelope glycoprotein and RNase, which is important for control of the innate immune response and persistent infection. The latter functions are connected with secretion of a certain amount of E^rns^ from the infected cell. Retention/secretion of E^rns^ is most likely controlled by its unusual membrane anchor, a long amphipathic helix attached in plane to the membrane. Here we present results of experiments conducted with a lipid vesicle sedimentation assay able to separate lipid-bound from unbound protein dissolved in the water phase. Using this technique we show that a protein composed of tag sequences and the carboxyterminal 65 residues of E^rns^ binds specifically to membrane vesicles with a clear preference for compositions containing negatively charged lipids. Mutations disturbing the helical folding and/or amphipathic character of the anchor as well as diverse truncations and exchange of amino acids important for intracellular retention of E^rns^ had no or only small effects on the proteins membrane binding. This result contrasts the dramatically increased secretion rates observed for E^rns^ proteins with equivalent mutations within cells. Accordingly, the ratio of secreted versus cell retained E^rns^ is not determined by the lipid affinity of the membrane anchor.

## Introduction

Pestiviruses are among the most important pathogens of livestock farming [1]. Four virus species are known, two types of bovine viral diarrhea virus (BVDV^1^), classical swine fever virus (CSFV) and border disease virus (BDV) of sheep belong to the genus *Pestivirus* that is grouped in the family *Flaviviridae* [2]. The family also comprises the genera *Flavivirus* with the type species yellow fever virus, *Hepacivirus* with the type species human hepatitis C virus, and *Pegivirus* including GB virus C and a variety of pegiviruses from different host species. All these viruses are enveloped and share basic molecular features such as a single stranded RNA genome of positive polarity containing one long open reading frame (ORF). The ORF is translated into a polyprotein that is cleaved into the mature viral proteins by host cellular and viral proteases [3].

On the molecular level, pestiviruses share significant similarity with human hepatitis C virus. Especially the organization of the non-structural proteins in the polyprotein and their basic biochemical features as well as principal functions show striking parallels. However, major differences are found within the 5’ terminal quarter of the genome. The pestiviral RNA encodes two proteins, N^pro^ and E^rns^, in this region which are missing in HCV, whereas the latter virus expresses the F’ protein from a second reading frame overlapping the long ORF [3].

N^pro^ and E^rns^ of pestiviruses are involved in blocking the type 1 interferon response of the host. N^pro^ is a non-structural protein encoded by the 5’ terminal fragment of the ORF. It induces degradation of the interferon regulatory factor 3 (IRF3) via the proteasome, thereby blocking the IFN-1 response of the infected host cell [4–13]. E^rns^ belongs to a group of three glycosylated structural proteins located on the envelope of pestivirus particles [14]. All of these three proteins are necessary for production of infectious viruses. E^rns^ is especially interesting since it is not only a structural component of the virion but has also RNase activity which is a unique feature among viral proteins [15, 16]. The E^rns^ RNase belongs to the family of T2-RNases, a group of ancient RNases widely distributed among eukaryotic species, the function of which is, however, not well understood [17–19]. In pestiviruses, the RNase activity was shown not to be essential for virus replication in tissue culture cells [20–22]. Inactivation of the enzyme by mutation led to virus mutants exhibiting almost wild type replication efficiency but showing strong attenuation in the natural host [21, 22]. The E^rns^ RNase was shown to be involved in blocking the IFN-1 response in bovine fetuses thereby contributing to establishment and maintenance of persistent infections [23]. Moreover, it has been shown recently that the IFN-α release from plasmacytoid dendritic cells (PDC) that came in contact with virus infected cells was blocked by the E^rns^ RNase [24].

A certain amount of E^rns^ is secreted from the infected cell and distributed in the infected animal via the blood stream [25–29]. The secreted protein was shown to block the cellular response to extracellular RNA [26, 27, 29, 30]. Moreover, secreted E^rns^ is internalized by cells and stays in the endosome in active form for several days. The internalized protein is able to block the dsRNA induced Toll-like receptor mediated IFN-1 response of these cells [31].

At least most hypotheses on the mechanism behind the E^rns^ virulence factor function focus on the secreted protein. The molecular basis for the equilibrium between secretion and retention of E^rns^ still is not well understood. This equilibrium should be important for pestivirus biology, since increased secretion would reduce the amount of Erns available for virion production, whereas complete intracellular retention of the RNase would impair the ability to counteract the host’s interferon response to pestivirus infection. We have shown before that E^rns^ is bound to membranes by a long amphipathic helix established from its C-terminal ~65 amino acids [25, 29, 32, 33]. A variety of mutations were found to increase secretion of E^rns^ and reduce its recovery from the membrane fraction. The obtained results were in frame with the hypothesis that the amphipathic character of the membrane anchor is important for binding to intracellular membranes and retention within the cell [25, 29]. However, it was unclear whether the observed increased secretion is due to a generally reduced affinity of the anchor for lipid membranes or resulted from other mechanisms. Here, we report on experiments conducted with a simple *in vitro* membrane binding assay based on sedimentation of test proteins with lipid vesicles of different compositions. Using this test system we analyzed the effect of different E^rns^ anchor mutations on lipid binding and compared the results with data on secretion/retention of the equivalent mutants obtained upon transient expression in cells.

## Material and Methods

### Cells and viruses

BHK-21 cells (kindly provided by T. Rümenapf) were grown in Dulbecco’s modified Eagle’s medium supplemented with 10% fetal calf serum and nonessential amino acids. The modified vaccinia virus strain Ankara containing the phage T7 RNA polymerase (MVA-T7) [34] was kindly provided by B. Moss (National Institutes of Health, Bethesda, Md.).

### Construction of recombinant plasmids

QuikChange mutagenesis (Stratagene, Heidelberg, Germany) was employed according to the supplier’s instructions to introduce substitutions, insertions or deletions. The constructs SSeqE^rns^-E1 and N^pro^-E^rns^ served as templates for all mutagenesis approaches for characterisation of the E^rns^/E1 cleavage site.

The cloned PCR products were all verified by nucleotide sequencing with the BigDye Terminator Cycle Sequencing Kit (PE Applied Biosystems, Weiterstadt, Germany). Sequence analysis and alignments were done with Geneious software (Biomatters, Auckland, New Zealand).

Further details of the cloning procedures and the sequences of the primers used for cloning and mutagenesis are available on request.

### Expression, metabolic labeling, and immunoprecipitation of proteins

Transient expression of plasmids in BHK-21 cells using vaccinia virus MVA-T7 was done as described [29]. For radioactive labelling of proteins, the cells were washed twice with label medium (without cysteine and methionine) 4 h after transfection and incubated in this medium for 1 h. Afterward, the medium was replaced by the label medium containing 0.1 mCi/ml of [35] Met-label (Hartmann Analytic GmbH, Braunschweig, Germany), and the cells were incubated for another 16–20 h at 37°C. The medium was removed and used to detect secreted proteins. Labelled cells were washed twice with phosphate-buffered saline (PBS) and frozen within the dishes. The cell extracts were prepared in buffer RIPA (20mM Tris, 100mM NaCl, 1mM EDTA, 1% Triton X-100, 0.1% DOC, 0.1% SDS, 2 mg/l BSA, pH 7.6). 500 μl of cell extract or supernatant were mixed with 50 μl 10% SDS denatured at 95°C, and sonicated for 20 sec (Branson Sonifier B15, water bath 100 W). Insoluble debris was pelleted at 5,000 rpm and the supernatant further clarified at 45,000 rpm in a TLA55 rotor (Beckmann Coulter, Krefeld, Germany) at 4°C. The cell extracts and the supernatant were incubated with 5 μl of monoclonal antibody WB210 (CCPro, Oberdorla, Germany). Before precipitation the antibodies were crosslinked with rabbit anti-mouse serum (Dianova, Hamburg, Germany). Precipitates were formed with cross-linked Staphylococcus aureus.

Analysis of the precipitated proteins was done by SDS-PAGE using Tricine-buffered gels [35]. Following electrophoresis, the gels were fixed for 1 h with an aqueous solution of 30% methanol and 10% acetic acid, rinsed for 3 h in water containing 20% methanol and 3% glycerol, vacuum-dried at 60°C, and exposed to BioMax X-ray films (Kodak, Stuttgart, Germany). Alternatively, quantification of the precipitation products was done with a phosphorimager (Fujifilm imaging plate [Raytest, Straubenhardt, Germany] and Fujifilm BAS-1500 phosphorimager [Raytest]). Computer-aided determination of the intensities of the respective signals was carried out with AIDA Advanced Image Data Analyzer Software, v4.19.029 (Raytest). The statistical evaluation of the results was done as described [29].

### Expression and purification of proteins

The expression of proteins in E.coli strain BL21(DE3) grown in standard LB-Medium was done essentially as described before [32]. Briefly, 1l of medium was inoculated with an overnight culture until the OD600 of the mixture was between 0.05 and 0.1. The bacteria were grown at 37°C and 220 rpm to an OD600 of 0.8. Protein expression was induced by addition of IPTG (final concentration 0.5 mM). The bacteria were incubated at 20°C and 220 rpm and harvested after 3h by centrifugation for 10 min at 5000 x g and 4°C. Lysis was done after resuspension in 15 ml Lysisbuffer [50 mM NaH_2_PO_4_ pH 8.0, 300 mM NaCl, 30 mM Imidazol, Lysozym, 6% TritonX-100, 1 tab. Roche Complete Protease inhibitor without EDTA (Roche, Mannheim, Germany)]. After 10 min incubation at room temperature three freeze-thaw cycles were performed with liquid nitrogen and warm water followed by sonication for 6 x 30 sec on ice (Branson Sonifier B15, level 7, cycle 80%). The insoluble debris was removed by centrifugation (Beckman JA17 rotor, 30 min, 31,000 x g) at 4°C.

The purification was started with a 5 ml Ni-NTA column (Protino Ni-NTA Columns, Macherey-Nagel, Germany) on an FPLC system (LKB GradiFrac, Pharmacia Biotech, Freiburg, Germany) with a flow rate of 3 ml/min. UV absorbance at 280 nm was measured with a connected absorbance recorder (LKB Optical Unit, LKB REC102, Pharmacia Biotech) to identify protein containing fractions. A step gradient of 50 mM and 100 mM imidazole was used to prevent unspecific protein binding and elution was accomplished with 300 mM imidazole. Afterwards, the protein containing fractions were pooled and ultrafiltrated (Amicon Ultra-15, Millipore). The retentate was diluted in lipid assay buffer (10 mM KH_2_PO_4_, 100 mM NaCl, 2,7 mM KCl) [36] and ultrafiltrated again until the Imidazol concentration was less than 1 mM.

### Lipid pull-down assay

Vesicles were generated essentially as described before. In brief, the appropriate amount of each lipid (Avanti Polar Lipid) first wassuspended separately in lipid assay buffer (10 mM KH_2_PO_4_, 100 mM NaCl, 2,7 mM KCl) to a concentration of 25 mg/ml. These suspensions were sonified according to the suppliers protocol [37, 38] with slight modifications (Branson Sonifier B15, water bath, 100 W at 38°C [36]) until the lipids were completely dissolved. After cooling to room temperature the different lipid solutions were mixed to obtain a total volume of 50 μl per assay with the desired ratio of the individual lipids. These mixtures were sonified for 2 x 30 sec as described above, to obtain vesicles with mixed lipids. According to the suppliers description, the resulting vesicles have a size of 15 to 50 nm which is in the range of pestivirus particles (40 to 60 nm diameter including the envelope protein layer) to the envelope membrane of which E^rns^ is bound via its C-terminal amphipathic helix.

The purified test protein was dissolved in lipid assay buffer to 120 nmol/ml by sonification [36]. 50 μl of this solution were added to 50 μl lipid vesicle solution. The mixture was immediately mixed by pipetting and then incubated at room temperature for 5 min. Sedimentation of the vesicles was done at 20°C for 30 min and 100,000 g in a table top ultracentrifuge (Beckman-Coulter, Krefeld, Germany) as described before [36]. 20 μl of the supernatant were transferred into a fresh tube and mixed with 10 μl SDS sample buffer (supernatant sample). The rest of the supernantant was discarded. The pellet was washed with 100 μl lipid assay buffer and centrifuged for 5 min at ca. 20,000 xg to remove unbound protein. The supernatant was again discarded, the pellet resuspended in 20 μl SDS sample buffer, heated for 5 min to 95°C, and vortexed immediately. The solution was diluted with 60 μl water, vortexed, and centrifuged again for 5 min at about 20,000 xg to reduce the amount of lipids in the sample which hampers the following SDS-PAGE. 20 μl of the supernatant were transferred to a fresh tube (pellet sample). 8 μl of the pellet samples and 15 μl of the supernatant samples (10% of the total amount of each sample) were analyzed by 18% SDS-PAGE and the gel was Coomassie stained. The protein bands were quantified densitometrically using the program “UNScan-IT”. The percentage of the lipid vesicle bound protein was calculated by division of the value determined for the protein in pellet by the sum of the values for the protein in pellet and supernatant and multiplied by 100. For each protein the mean value of three independent experiments was determined and given in the graphs together with the standard deviation.

Lipids used for the experiments:

DMPA (PA): 1,2-dimyristoyl-sn-glycero-3-phosphatidylacid
DMPC (PC): 1,2-dimyristoyl-sn-glycero-3-phosphatidylcholine
DMPE (PE): 1,2-dimyristoyl-sn-glycero-3-phosphatidylethanolamin
DMPG (PG): 1,2-dimyristoyl-sn-glycero-3-phosphatidylglycerol
DMPS (PS): 1,2-dimyristoyl-sn-glycero-3-phosphatidylserine
PI: Phosphatidylinositol
Sph: Sphingomyelin (from hen egg white)

Lipids were purchased from Avanti Polar Lipids, Alabaster, Alabama, USA

## Results

### Establishment of a test system for binding of the E^rns^ membrane anchor to lipid vesicles of different composition

**Fig 1:**
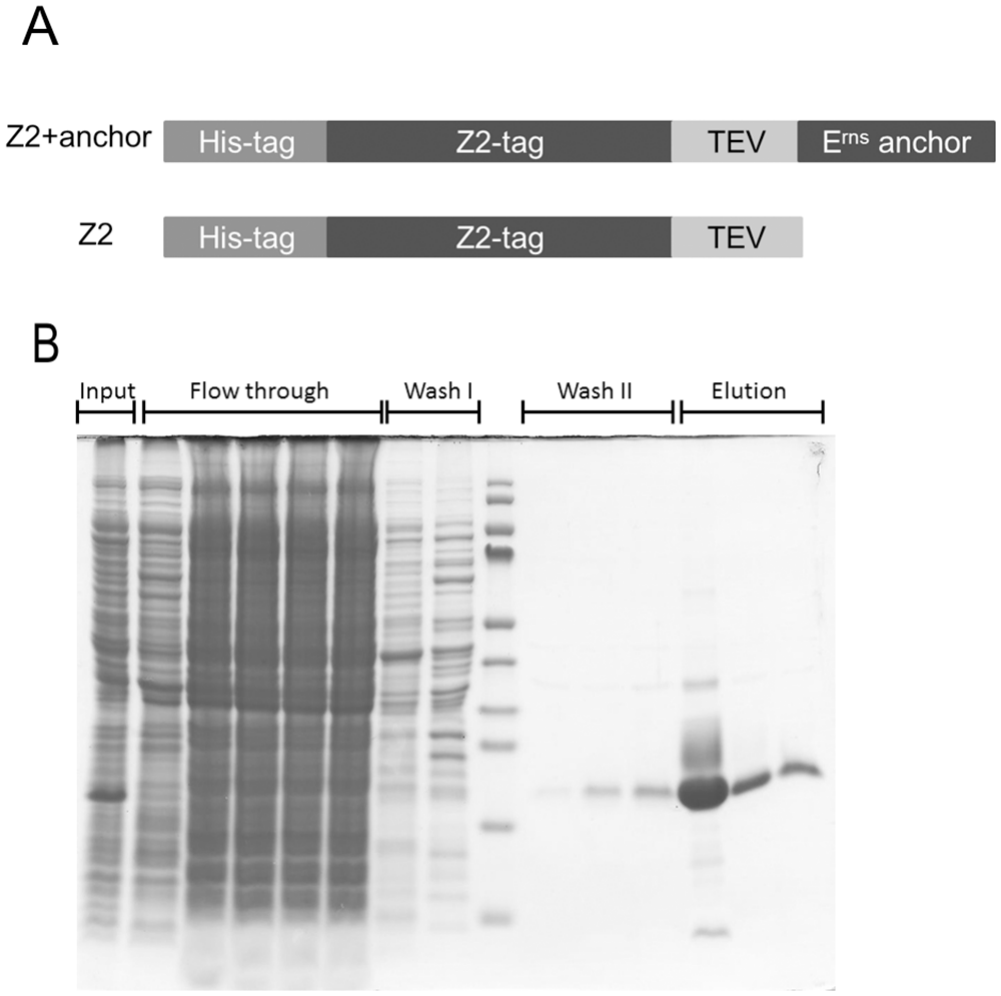
Test proteins for lipid pull down assay. (A) Schematic representation of the composition of the test proteins Z2+anchor and Z2 control. His-tag: 6xHis residues; Z2-tag: single Z-domain from *S*. *aureus* protein A; TEV: tobacco etch virus proteinase cleavage site; E^rns^ anchor: 65 C-terminal residues of the E^rns^ protein from BVDV CP7 [41]. (B) Coomassie-stained SDS-PAGE with samples taken during expression and purification of Z2+anchor. The individual fractions analyzed here are given on top. Between Wash I and Wash II, a protein size marker (PageRuler prestained protein ladder, New England Biolabs, Schwalbach, Germany) is shown (bands represent sizes of 170, 130, 100, 70, 55, 40, 35, 25, 15, 10 kDa, top to bottom).

The pestivirus E^rns^ protein is translocated through the ER membrane into the ER during translation. The signal sequence inducing translocation is cleaved off and the resulting protein that is deficient of a transmembrane region or other standard membrane anchor is bound to the inner side of the ER membrane by a long C-terminal amphipathic helix [25, 32, 33, 39]. A certain amount of the protein synthesized within infected or transfected cells is secreted into the cell free supernatant. The percentage of secreted protein increases dramatically upon mutations within the C-terminal region of the protein, especially when these mutations disturb the helical structure or the amphipathic character of the membrane anchor [39]. In a simple model, this finding could be interpreted in a way that E^rns^ is secreted when not bound to a membrane so that weakened membrane binding by a mutated anchor would result in increased E^rns^ secretion. This model would imply that intracellular retention of the protein can only be achieved when it is bound to the membrane and thereby has contact to some unknown cellular (membrane) factor determining its intracellular localization. In contrast, not membrane bound E^rns^ would be transported and secreted with the bulk flow. This attractive model is challenged by the fact that the E^rns^ sequence supposed to interact with the lipid bilayer consists of more than 50 amino acids which raises the question why single site mutations or single residue insertions/deletions should have such profound consequences. It therefore had to be questioned whether the mere reduction of lipid binding affinity resulting from these mutations could be responsible for the observed increase in secretion. We established a simple *in vitro* lipid pull-down assay to study the effect of such changes in the absence of cellular factors. We used the construct Z2+anchor that is equivalent to pd29G [32] for bacterial expression. The expressed protein is composed of a 6xHis tag, a Z2-tag (single Z-domain from S. aureus protein A), a tobacco etch virus (TEV) proteinase cleavage site, and the 65 C-terminal residues of the E^rns^ protein from BVDV CP7 (Fig 1A) [32, 40]. The two tags allow purification of protein and increase its solubility whereas the TEV cleavage site serves as a linker to separate tags and E^rns^ membrane anchor. As a control, a Z2 construct was established that is equivalent to Z2+anchor but lacks the viral sequence. The proteins encoded by the two constructs were expressed in E.coli and purified via Ni-NTA chromatography (Fig 1B). 6 nMol of protein were used for a standard lipid pull-down assay.

The pull-down assay relied on the separation of protein bound to lipid vesicles from protein dissolved in the aqueous phase via sedimentation [36]. Vesicles composed of different lipids were generated and mixed with the aqueous solution containing the desired protein. After a short incubation time, the vesicles were pelleted by ultracentrifugation, washed and analyzed by SDS PAGE together with a sample of the supernatant of the ultracentrifugation step.

**Fig 2:**
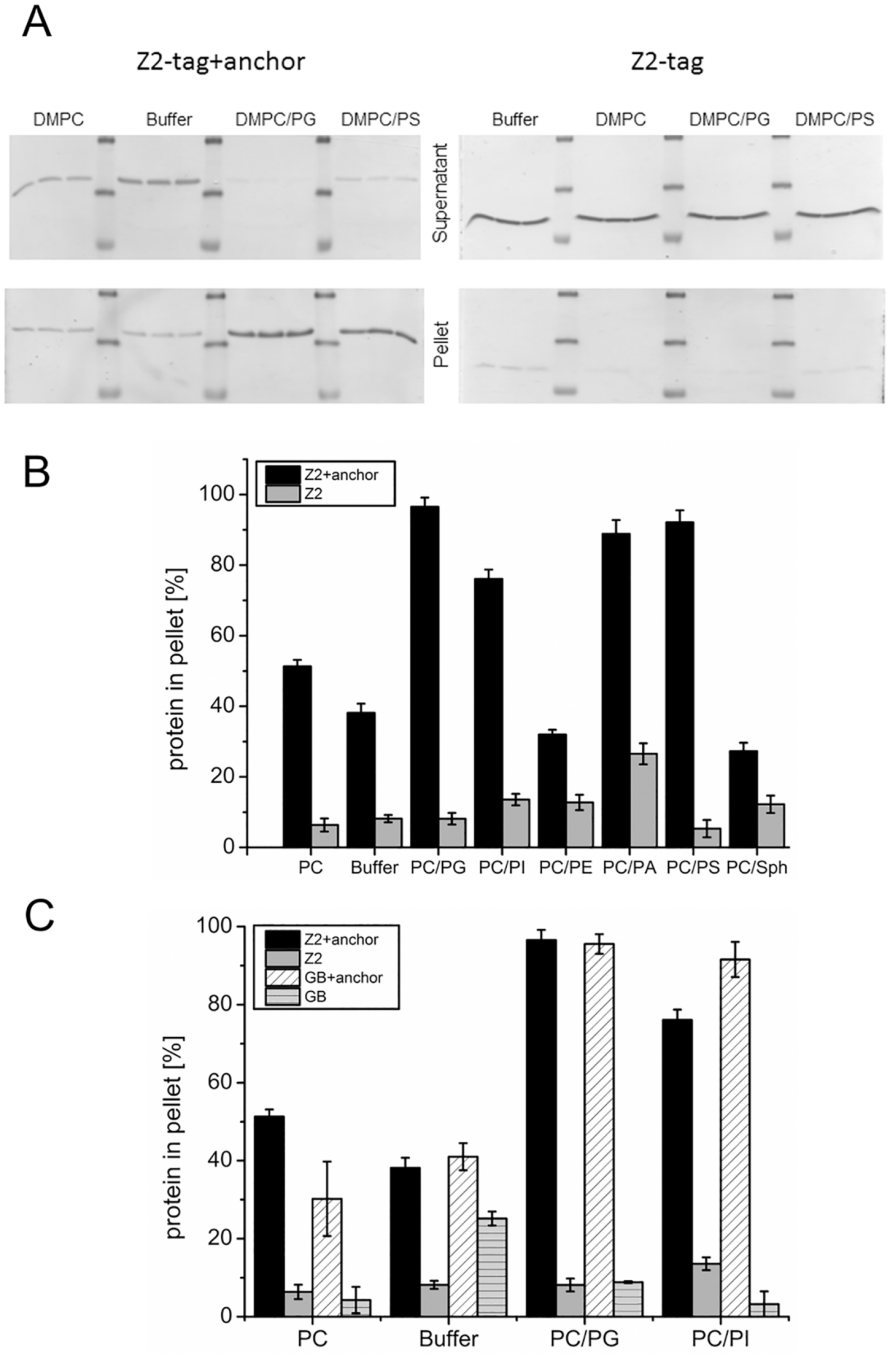
Lipid pull down assay. (A) Coomassie-stained SDS-PAGE with the proteins found in the supernatant (upper part) or pellet (lower part) of pull down assays conducted in buffer or in buffer containing lipid vesicles of different compositions (composition indicated on top: DMPC/PG = 95% DMPC/ 5% DMPG; DMPC/PS = 95% DMPC/ 5% DMPS). Left part: protein Z2+anchor was used in pulldown; right part: Z2 control. For the quantification given in B and C the total amount of the detected protein was determined densitometrically and the percentage of the product found in the pellet fraction was calculated. Accordingly, the percentage of Z2+anchor in the pellet is increased in the presence of DMPC vesicles as the amount of protein in the supernatant is lower than in buffer alone (left part of left gel). (B) Quantitative results of pull down of Z2+anchor (black bars) or Z2 control (gray bars) with vesicles composed of different lipids as indicated below the X-axis. PC: 100% DMPC; Mixtures: 95% DMPC and 5% of DMPG (PC/PG), L-α-Phosphatidylinositol (PC/PI); DMPE (PC/PE), DMPA (PC/PA), DMPS (PC/PS) or sphingomyelin (PC/Sph). Buffer: control of lipid binding buffer without lipids. (C) Comparison of binding of proteins containing the Z2 tag or an alternative tag (GB = GB-carrier protein [42]) to vesicles with the indicated lipid composition or buffer without lipids. The bars in (B) and (C) give the percent of the total protein recovered in the pellet fractions and represent the mean of at least three independent experiments with the standard deviation shown by error bars.

As a first step, binding of Z2+anchor to different lipids was tested. Since phosphatidylcholine represents the most abundant lipid in biological membranes, dimyristoylphosphatidylcholine (DMPC) alone or mixtures of 95% DMPC and 5% of another lipid were used in the experiments (Fig 2). About 40% of the Z2+anchor protein was found in the pellet when the experiment was conducted in buffer without any lipid which served as a negative control. In contrast, only about 10% of Z2 was recovered from the pellet. This difference is due to the reduced solubility of Z2+anchor protein which results in certain amounts of aggregates thatare pelleted during ultracentrifugation. Addition of lipids should increase solubility of hydrophobic test proteins because free lipids that are not engaged in vesicle formation act as a detergent. Importantly, the detection of a higher percentage of pelleted material in the buffer vesicle mixture versus buffer alone demonstrates specific pelleting of the protein with the vesicles. In suspensions with DMPC vesicles the ratio between pelleted versus soluble Z2+anchor changes resulting in ~10% increase of this protein in the pellet fraction, while even a slight decrease in pelleted protein was detected for the Z2 protein in the presence of DMPC vesicles. Mixture of the two zwitterionic lipids DMPC and DMPE did not increase recovery of the test protein in the pellet (Fig 2B). A similar result was also seen for DMPC mixed with sphingosine. In contrast, mixtures containing the negatively charged lipids DMPG or DMPS in addition to DMPC were able to bind Z2+anchor much more efficiently, so that 80- to almost 100% of the protein were found in the pellet (Fig 2A and 2B). This result can be explained by the fact that the E^rns^ membrane anchor contains a net charge of +2 with a concentration of positively charged amino acids in the C-terminal region. Earlier work had shown that the amount of helical folding of the anchor was higher in a mixture of DMPC and DMPG than in DMPC alone [25, 32], and in the binding assay this combination showed the highest recovery rate in the pellet.

To exclude that the N-terminal tag sequence influences the lipid binding of the E^rns^ membrane anchor a construct, in which the Z2 tag was replaced by the GB-carrier protein [42] was tested in an equivalent experiment which did not yield significantly different results (Fig 2C). Thus, it can be concluded that the E^rns^ membrane anchor binds specifically to lipid vesicles with a preference for lipid mixtures containing negatively charged head groups.

### Membrane binding of the E^rns^ anchor at different temperatures and incubation times

**Fig 3:**
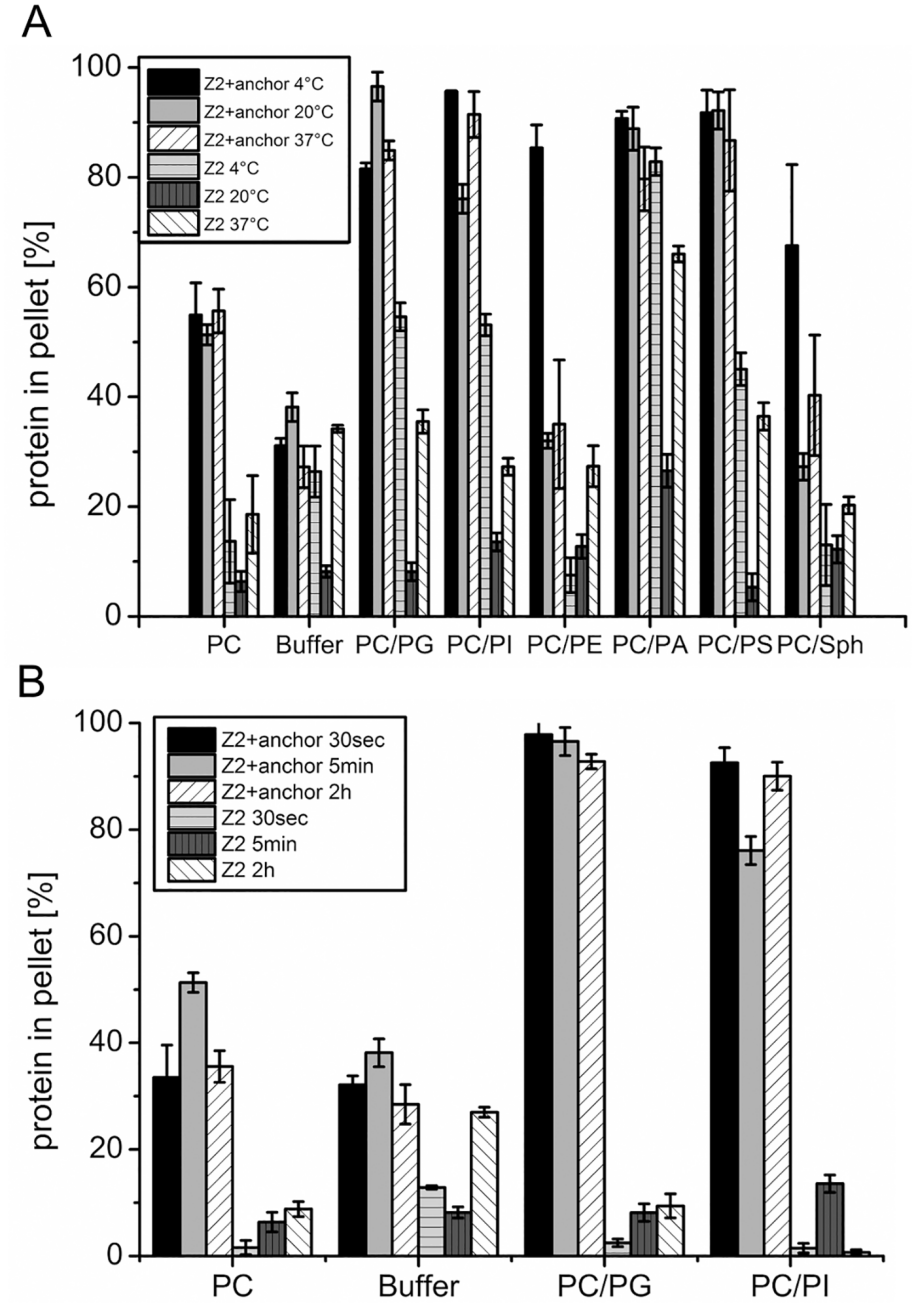
Temperature dependence and kinetics of lipid binding of the E^rns^. (A) Temperature dependence and (B) kinetics of lipid binding of the Z+anchor compared with Z control. The composition of the lipid vesicles is indicated below the X-axis and specified in detail in the legend to Fig 2B.

The fluidity of lipid membranes is highly dependent on temperature, and accordingly the interaction of a lipid binding protein with a membrane can vary considerably with temperature. We again tested different lipid compositions and incubated the vesicles at 4°C, 20°C and 37°C with Z2+anchor for 5 minutes before ultracentrifugation at this temperature. Z2 alone again served as a control. Temperature had little influence on anchor binding to vesicles composed of PC or PC/PG, PC/PI, PC/PA or PC/PS. Also the values determined for buffer without lipids were very similar for all tested temperatures. Significant differences were only found for PC/Sph and especially PC/PE mixtures where binding at 4°C was highly enhanced compared with the other two temperatures (Fig 3A). In contrast, the recovery of the Z2 tag without anchor showed considerable variation in several systems. In most cases, 4°C led to the highest recovery of Z2 followed by 37°C. In some of the assays the Z2 values at 4°C approached the levels obtained with Z2+anchor so that a high percentage of the observed recovery of the latter protein in the pellet should result from the tag and not the anchor sequence. Importantly, the difference between Z2+anchor and Z2 was highest at 20°C in all assays except the PC/PE and PC/Sph vesicles. Thus, the specificity of the binding assay was best at 20°C for the combinations buffer alone, DMPC vesicles, and vesicles composed of DMPC/DMPG or DMPC/DMPI. Accordingly, these conditions were used for the tests conducted later on.

It is not clear, what determines the fate of the E^rns^ protein within living cells. Secretion of the protein could result from delayed folding of the anchor, so that the kinetics of lipid assisted helical folding could control the secretion level of the protein. To receive an impression of the time needed for establishment of lipid binding we determined the recovery rate of Z2+anchor in vesicle pellets after 30 seconds, 5 minutes and 2 hours of incubation (Fig 3B). The results were very similar for all tested time points so that it can be concluded that establishment of lipid/anchor interaction is fast. The standard lipid binding assay was conducted with an incubation time of 5 minutes.

### Other amphipathic helices can replace the E^rns^ membrane anchor in in vitro lipid binding assays but not in cells

**Fig 4:**
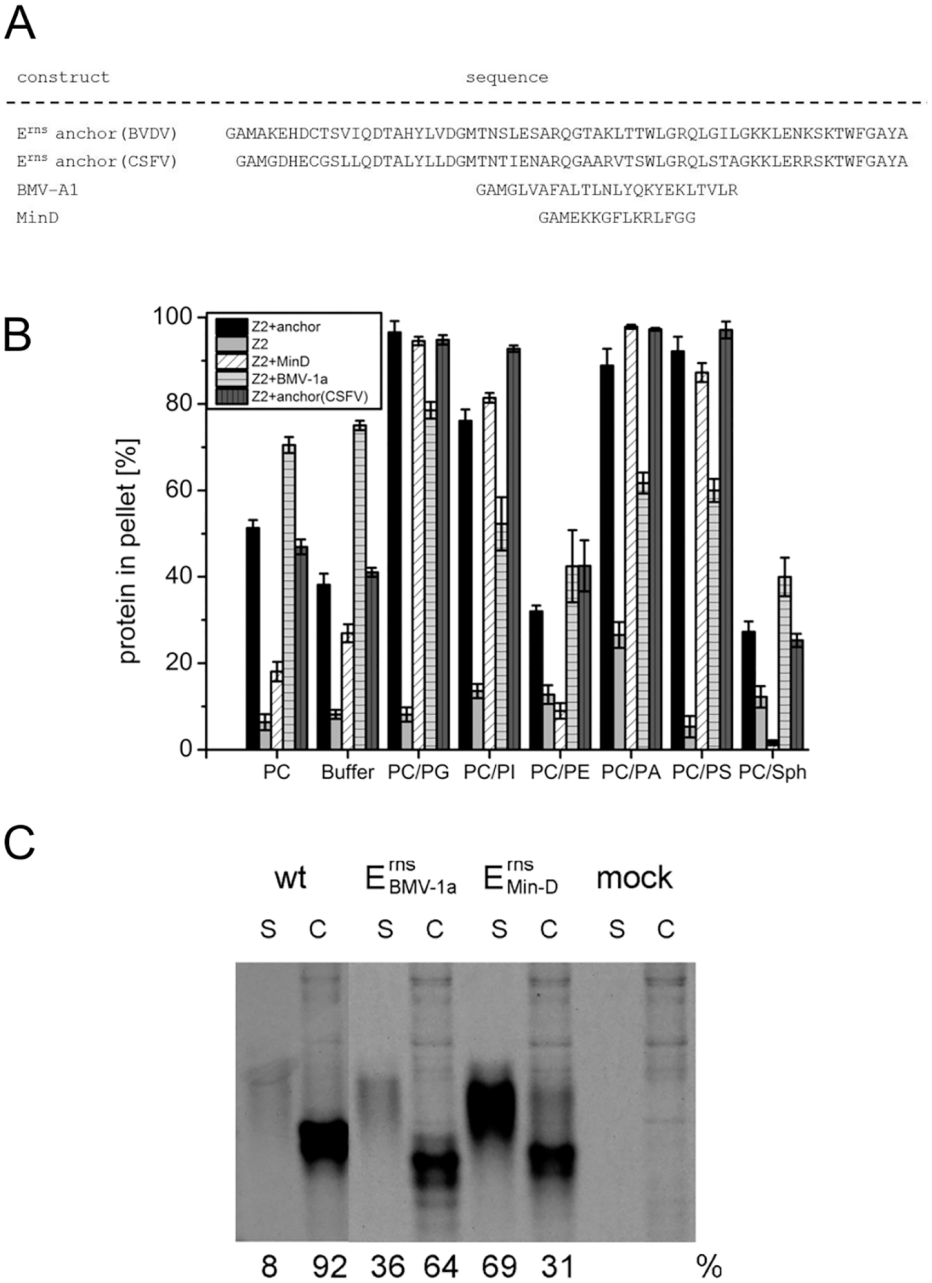
Comparison of lipid binding of constructs with Z2 tags fused to different amphipathic helixes. (A) Sequences of the tested amphipathic helices are given. E^rns^ anchor (BVDV) represents the sequence present in the standard Z2+anchor construct. Below, the corresponding sequence of the related classical swine fever virus E^rns^ protein is shown. BMV-1a represents the sequence from the brome mosaic virus 1a protein. MinD is the amphipathic helix sequence from the MinD protein. Please note that the BVDV E^rns^ anchor sequence contains a consensus site for N-glycosylation close to the carboxyterminus. This sequence is conserved for at least many BVDV but we have no indication that this potential site is glycosylated. Moreover, this motif is absent from the corresponding sequences of the closely related classical swine fever virus (CSFV) and border disease virus (BDV) that show equivalent features with regard to E^rns^ secretion as BVDV. Thus, the presence of this motif in BVDV likely is not relevant for E^rns^ membrane binding/secretion. (B) Presentation of the results of lipid pull down assays with Z2 constructs containing the anchor sequences given in (A). Vesicles with different lipid compositions were tested (see legend of Fig 2 for further information on lipids and vesicle compositions). The bars give the percentage of the total protein recovered in the pellet fractions and represent the mean of at least three independent experiments with the standard deviation shown by error bars. (C) Secretion/retention rates of E^rns^ proteins with different carboxyterminal amphipathic helices. As a reference, wildtype E^rns^ of BVDV strain CP7 is shown on the left (wt), next to the constructs with the E^rns^ anchor replaced by the BMV-1a or MinD sequence. Phosphoimager based quantification of the results of immunoprecipitation experiments are given below the gel. For each construct the calculated amounts of secreted and retained protein are given in percent of the total recovered protein (sum of the values determined for supernatant and cell extract). All constructs were tested at least three times and the average values are given. The considerably different electrophoretic mobility of E^rns^ from cell extracts and supernatant results from processing of the carbohydrate groups when the protein is transported through the Golgi apparatus during secretion. In the secreted form, more than 50% of the protein are made up by carbohydrates.

A variety of amphipathic helices serving as membrane binding domains of proteins have been described and analyzed in the past [43]. In order to compare membrane binding of already characterized sequences with the E^rns^ membrane anchor in our test system, two such sequences, the amphipathic helix of the *E*. *coli* MinD protein [44] and the membrane anchor of the 1a protein from brome mosaic virus (BMV-1a) [45] were used to replace the E^rns^ sequence in the Z2+anchor. Both foreign sequences were much shorter than the E^rns^ anchor (Fig 4A). In addition, the anchor sequence of an E^rns^ protein from classical swine fever virus (CSFV), a pestivirus belonging to another species and showing considerable sequence deviation from the BVDV protein, was tested. As expected, the CSFV anchor showed very similar characteristics as the BVDV anchor, since the biochemical similarity of the two sequences is high despite ~50% amino acid sequence divergence. However, also the MinD and BMV 1a sequences were able to hook the Z2 tag to the different lipid vesicles with very similar efficiency as the pestivirus sequences (Fig 4B). To test whether the MinD or BMV 1a sequences can substitute for the E^rns^ anchor with regard to retention in the cells, the anchor of CSFV E^rns^ was replaced by these sequences. Transient expression experiments revealed that almost 70% of the E^rns^/MindD protein was secreted into the cell free supernatant. For the BMV 1a construct approx. 36% secretion was determined which is still quite high compared to less than 10% for the wt protein (Fig 4C).

### The membrane anchor can tolerate significant disturbance of the amphipathic helix

**Fig 5:**
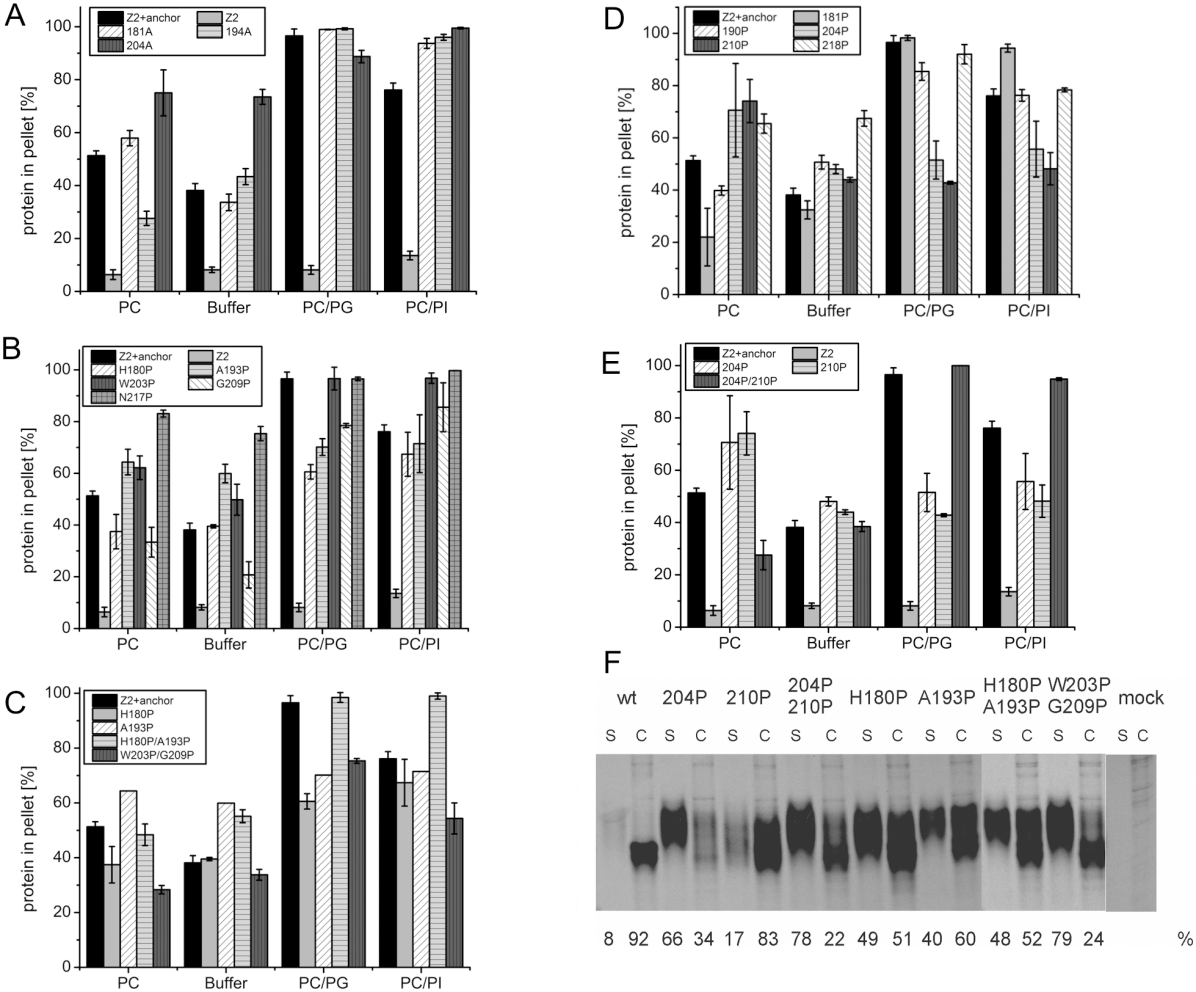
Effect of alterations affecting the amphipathic character of the E^rns^ membrane anchor on lipid binding *in vitro* and in cells. (A) Results of lipid pulldown assays with Z2+anchor constructs containing single alanine insertions downstream of positions 181, 194 or 204 (181A, 194A or 204A, respectively) to twist the helix, thereby interfering with the amphipathic conformation. (B) (C) Effects of replacement of individual amino acids in the anchor sequence by proline to interfere with helical folding. The individual exchanges are given in the graphs. (D) (E) Combination of the alterations tested in (A) and (B) (C) by insertion of proline residues into the anchor sequence. The prolines were inserted downstream of the positions indicated in the graphs. (A-E) show the results of lipid pull down assays with the mutated Z2 constructs tested with different lipid compositions as above (see legend of Fig 2 for further information on lipids and vesicle compositions). The bars give the percentage of the total protein recovered in the pellet fractions and represent the mean of at least three independent experiments with the standard deviation shown by error bars. (F) Secretion/retention rates of E^rns^ proteins with the different indicated mutations in the carboxyterminal amphipathic helix. As a reference, wildtype E^rns^ of BVDV CP7 is shown on the left (wt). Phosphoimager based quantification of the results of immunoprecipitation experiments are given below the gel. For each construct the calculated amounts of secreted and retained protein are given in percent of the total recovered protein (sum of the values determined for supernatant and cell extract). All constructs were tested at least three times and the average values are given. As pointed out in detail in the legend to Fig 4C, the increased molecular weight of secreted E^rns^ is due to carbohydrate processing.

Previous studies with transiently expressed proteins had shown that a variety of mutations disturbing the amphipathic helix via insertion of single amino acids [29] or replacement of a residue by the helix breaking amino acid proline (Tews and Meyers, unpublished) resulted in enhanced secretion of E^rns^. Importantly, the introduction of such changes at positions in the middle of the helical region had a much higher impact than the equivalent mutations close to the end of the helix. These findings proved the importance of the integrity of the amphipathic helix for E^rns^ membrane association and retention within the cell. We wondered whether the above mentioned mutations interfered with basal lipid binding of the anchor since the remaining unchanged parts of the helix were still quite long compared to e.g. the MinD or BMV 1a helices which confer membrane binding with only15 or 25 amino acids as shown above. We first tested the influence of insertions of a single alanine residue downstream of positions 181, 194 or 204 (mutants 181A, 194A or 204A, respectively) (Fig 5A). The effects of these changes on the interaction of Z2+anchor with the preferred binding partners DMPC/DMPG or DMPC/DMPI vesicles were rather small or even not significant. Vesicles consisting of DMPC alone showed increased binding of 181A and 204A. However, the latter result is difficult to interpret since strongly increased recovery from the pellet was also observed when the 204A mutant was tested in pure buffer indicating decreased solubility of the protein. Most importantly, a significantly decreased lipid binding in consequence of the massive disturbance of the amphipathic character by the ca. 120 degree twist of the helix could not be shown. This stands in marked contrast to the results obtained after expression of equivalent E^rns^ mutants in cells [29].

As a second step, we tested proline substitution mutants. The analyzed mutants were H180P, A193P, W203P, G209P, N217P, a combination of the mutations at positions 180 and 193 (H180P/A193P) and a second double mutant W203P/G209P. Mutations W203P, G209P and N217P did not influence vesicle binding significantly, whereas A193P and N217P showed reduced solubility as indicated by increased recovery from the pellet in pure buffer (Fig 5B and 5C). H180P and A193P revealed a somewhat reduced binding to DMPC/DMPG, but this effect was quite small. Similarly, the combination of these two changes had no significant effect on lipid binding. In contrast, the double mutant W203P/G209P showed decreased binding to PC/PG and PC/PI, although also this result was not dramatic.

To increase the structural change induced by the mutations we inserted proline residues so that a separation of the amphipathic surface into two twisted parts was combined with a disturbance of the helical folding. Proline residues were inserted downstream of positions 181, 194, 204, 210 or 218. Only the mutants 204P and 210P showed significantly changed binding characteristics with increased binding to DMPC vesicles but reduced affinity to the vesicles containing the negatively charged DMPG or DMPI lipids (Fig 5D and 5E). In contrast, a double mutant containing proline insertions at 204 and 210 (204P/210P) showed reduced binding to DMPC but increased binding to DMPC/DMPI vesicles, whereas no effect was observed for DMPC/DMPG indicating an increased specificity for negatively charged lipids.

To be able to compare the above described results with the secretion rate of the equivalent mutants in the cellular system we quantified the transiently expressed E^rns^ protein and a selected set of mutants thereof in supernatant and lysates of transiently transfected cells as described before [29]. Fig 5F shows the gel with the precipitated proteins, and below the gel the results of the quantification are given as percentage of E^rns^ detected (mean of at least three independent experiments). All 4 tested proline substitution mutants showed significantly enhanced E^rns^ secretion rates by a factor of about 8 (single mutants or H180P/A193P double mutant) to 15 (W203P/G209P double mutant) (Fig 5F, right part) thereby demonstrating a clearly different effect on mere lipid binding versus secretion from the cell. Similarly, all of the tested proline insertion variants showed significantly higher E^rns^ secretion than the wt. As expected in the light of earlier results [29], the insertion downstream of residue 204 (204P) had a more prominent effect than the insertion after position 210 (210P) which is quite close to the carboxyterminal end of the helix (Fig 5F, left part). The effect observed for the double mutant 204P/210P was about equal to the value determined for single mutant 204P, indicating that the second insertion more downstream could not add significantly to the secretion rate any more.

**Fig 6:**
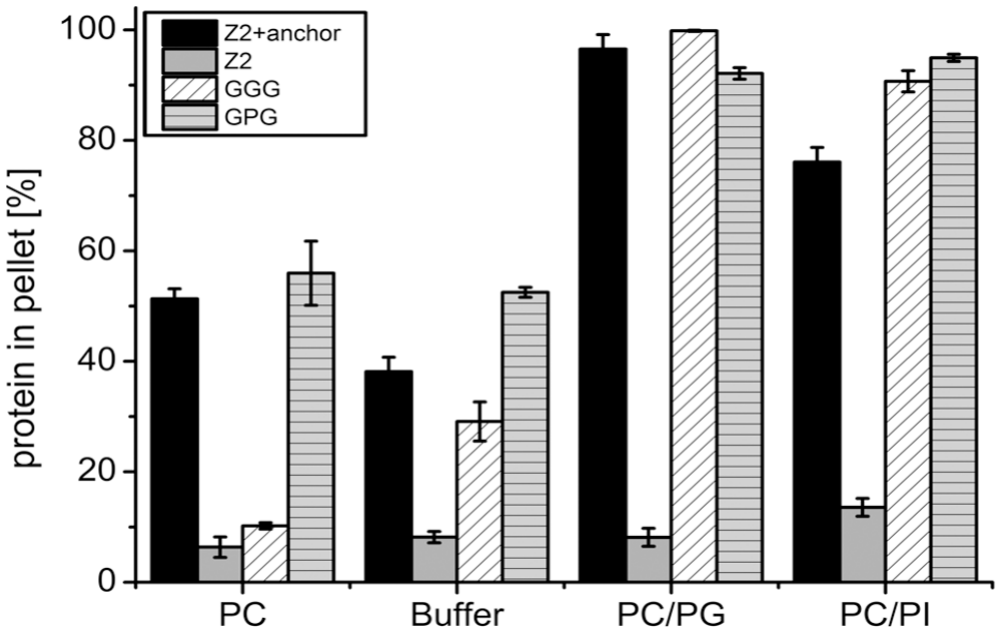
Effect of insertions in the amphipathic helix on lipid binding. 3 G residues (GGG) or the sequence GGGGPGGGGPGGGG (GPG) were inserted between residues 210 and 211 of the E^rns^ anchor. The insertions divide the amphipathic helix into an N-terminal and C-terminal part. The mutants were tested in lipid pull down assays with different lipid compositions as above (see legend of Fig 2 for further information on lipids and vesicle compositions). The bars give the percentage of the total protein recovered in the pellet fractions and represent the mean of at least three independent experiments with the standard deviation shown by error bars.

As a last step, we tested the influence of the insertion of 3 or 14 amino acids on lipid binding of the E^rns^ anchor. To this end, 3 glycine residues (mutant GGG) or the sequence GGGGPGGGGPGGGG (mutant GPG) were inserted between residues 210 and 211 thereby dividing the amphipathic helix into an N-terminal and C-terminal part. Since a helix turn contains approx. 3.6 amino acids, the angle between the two parts of the anchor encoded by these mutants with regard to the amphipathic faces is only small. Again, the change induced by these alterations with regard to the recovery rate in the pellet with vesicles composed of PC/PG or PC/PI was only small (Fig 6). The most striking observation was the strongly reduced binding of the GGG insertion mutant to DMPC vesicles indicating an increased specificity for negatively charged lipids.

Taken together, the results obtained with the various mutations affecting the integrity of the amphipathic helix proved that lipid binding of the E^rns^ membrane anchor can tolerate quite invasive changes which in the cell system resulted in grossly increased secretion of E^rns^ (this manuscript and [29])

### Shortened versions of the membrane anchor show reduced membrane binding

**Fig 7:**
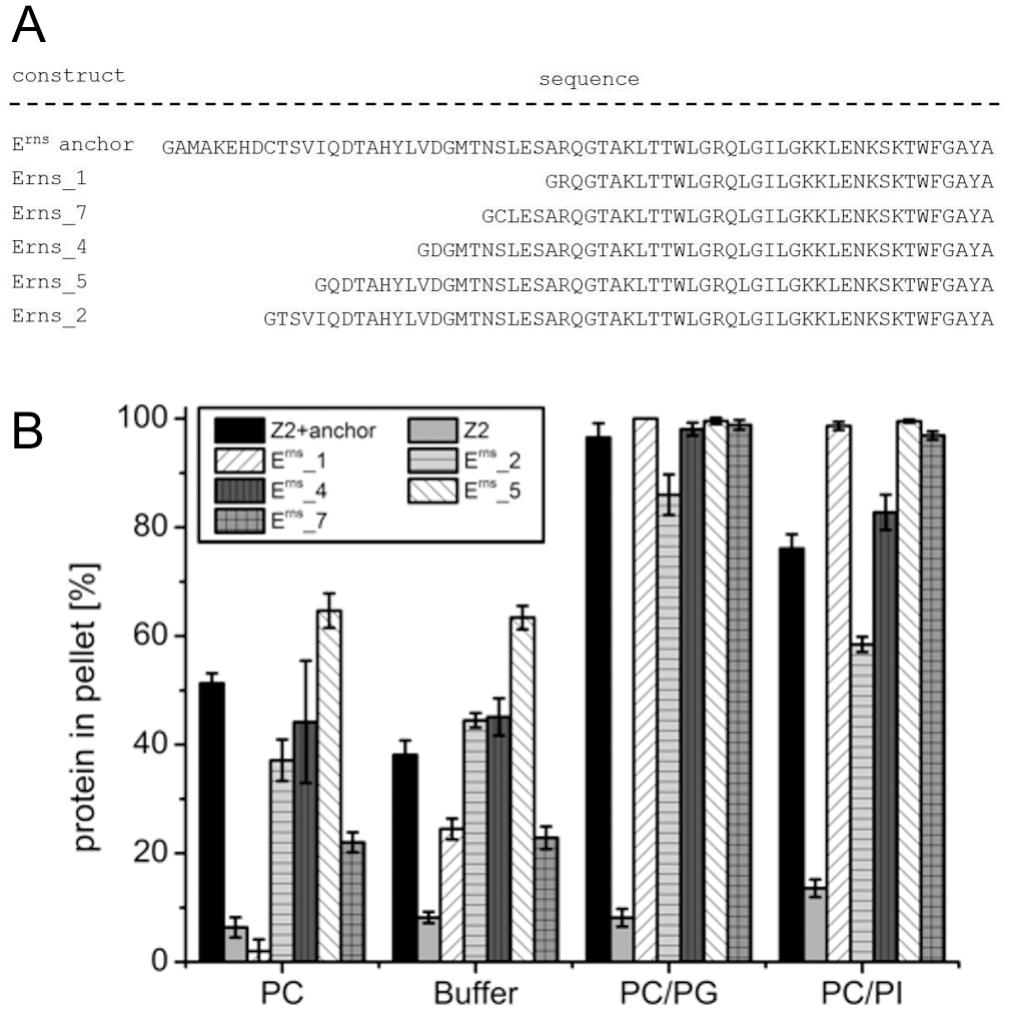
Influence of N-terminal truncation on lipid binding of the E^rns^ anchor. (A) Sequences of wt (E^rns^-anchor) and N-terminally truncated anchor sequences. (B) Results of lipid pull-down experiments with the constructs containing the sequences given in (A).

As already mentioned above, the E^rns^ amphipathic helix is surprisingly long for a membrane anchor compared to other sequences with the same function. We were therefore interested to see whether truncation of the anchor sequence would impair membrane binding. We first tested 5 N-terminally truncated versions of the viral sequence in the Z2 context (Fig 7A). Interestingly, only the versions with the shortest truncations showed somewhat altered lipid binding characteristics with overall reduced recovery in the vesicle pellet fraction (E^rns^_2) or increased recovery rates in all tested mixtures (E^rns^_5), whereas the proteins with the larger truncations tended to exhibit higher specificity for vesicles containing negatively charged lipids due to lower affinity to DMPC vesicles and equivalent or even higher recovery rates with vesicles containing negatively charged lipids (Fig 7B).

**Fig 8:**
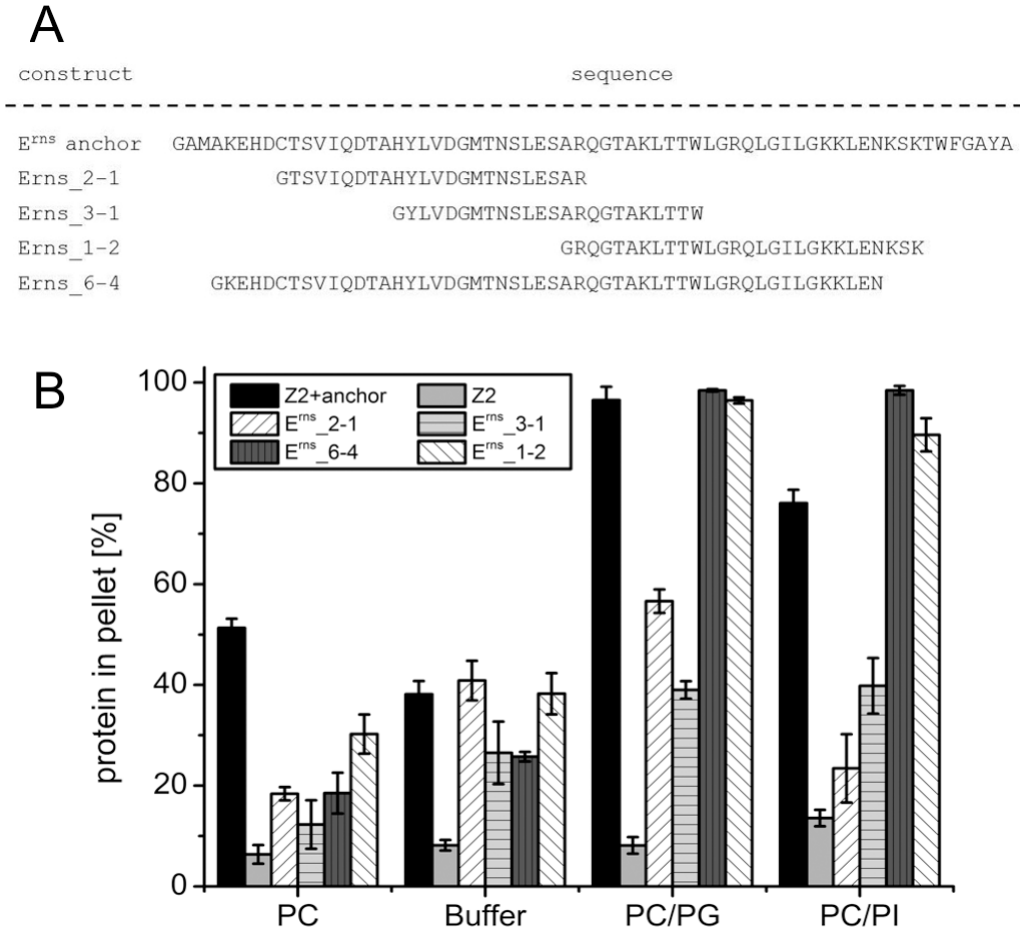
Lipid binding efficiency of internal fragments of the E^rns^ membrane anchor. (A) Sequences of wt (E^rns^-anchor) and N-terminally truncated anchor sequences. (B) Results of lipid pull-down experiments with the constructs containing the sequences given in (A).

As a further step we tested an anchor sequence construct with a C-terminal truncation of 10 residues named E^rns^_6–4 that is also N-terminally shortened by 3 residues compared to wt (Fig 8). Binding of this protein to DMPC/DMPG vesicles was equivalent to the wt anchor, whereas increased recovery was observed with DMPC/DMPI vesicles. In general, binding specificity to vesicles with negatively charged lipids was increased compared to the wt, since binding to DMPC alone was lowered to less than 50% of the wt level (Fig 8B). Thus, loss of 10 C-terminal residues could be tolerated very well, which stands in marked contrast to the data obtained with E^rns^ in cells where truncation by 4 residues already resulted in a dramatic increase in E^rns^ secretion [33].

In another approach, the anchor was divided into 3 internal overlapping parts which were tested separately for lipid binding (Fig 8). Construct E^rns^_2-1encoded a protein in which residues 172 to 194 of E^rns^ were fused to the Z2-tag, whereas the proteins derived from E^rns^_3–1 and E^rns^_1–2 contained residues 181–203 and 194–220, respectively. The N-terminal and middle fragments E^rns^_2–1 and E^rns^_3–1 showed severely reduced pellet recovery rates for all lipid vesicle compositions. Since precipitation in lipid free buffer was quite high for these proteins, their specific binding to lipids seems to be at least low. In contrast, the C-terminal fragment of the anchor introduced into E^rns^_1–2 conferred wt binding efficiency to DMPC/DMPG vesicles and only a rather slight reduction for DMPC/DMPI, whereas pelleting with vesicles composed of only DMPC was reduced to less than 20% of the wt level. Thus, it can be concluded that the C-terminal part of the E^rns^ anchor represents the major determinant for specific lipid binding. This part of the membrane anchor contains a high density of basic amino acids which is probably responsible for the higher affinity to lipids with a negative net charge.

**Fig 9:**
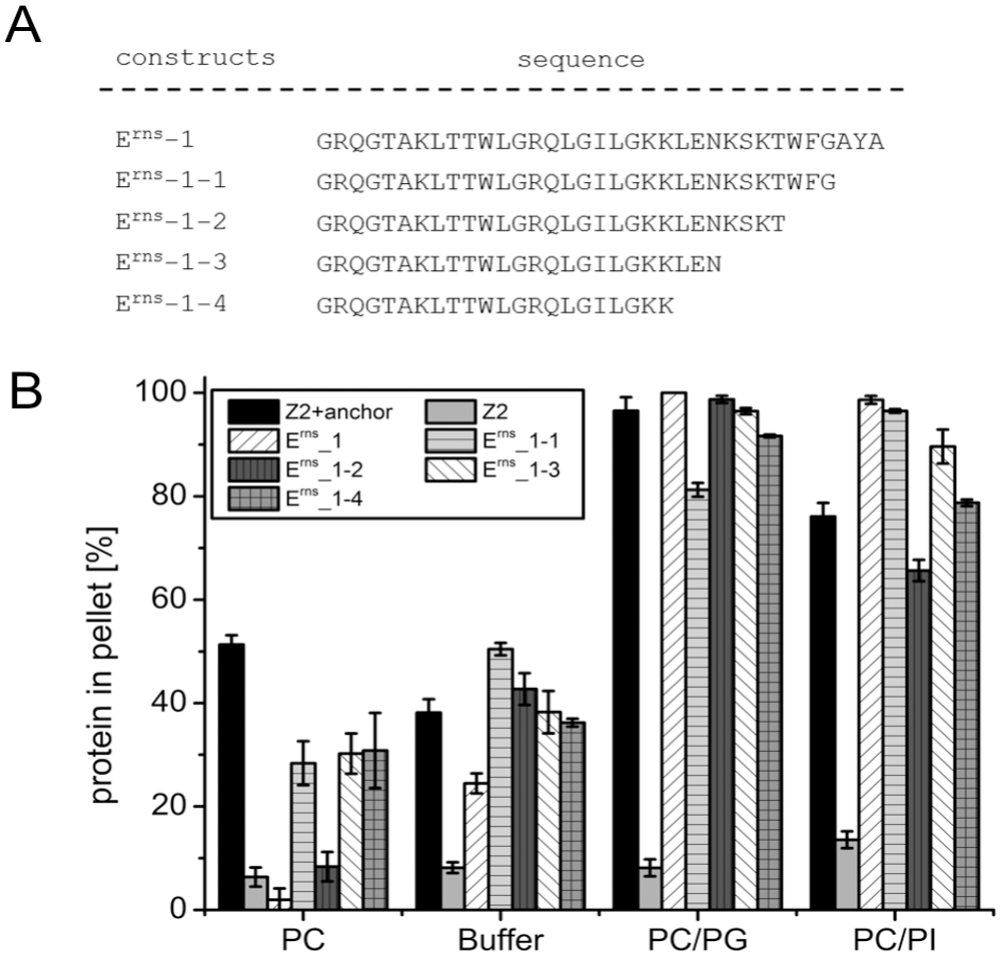
Effect of C-terminal truncation on lipid binding of an E^rns^ anchor fragment. (A) Sequences of the E^rns^-anchor fragment E^rns^-1 (C-terminal 35 amino acids of the anchor) and C-terminally truncated forms thereof. (B) Results of lipid pull-down experiments with the constructs containing the sequences given in (A).

Based on the N-terminal truncation product E^rns^-1 further C-terminally truncated protein mutants were tested for their binding efficiency to lipid vesicles (Fig 9A). We deleted up to 13 C-terminal residues of E^rns^-1 without significant effects on DMPC/DMPG binding and only slight effects on DMPC/DMPI interaction (Fig 9B) so that the major lipid binding domain of the E^rns^ anchor must be located within the sequence GRQGTAKLTTWLGRQLGILGKK (positions 194–215).

### Membrane binding and intracellular localization

**Fig 10:**
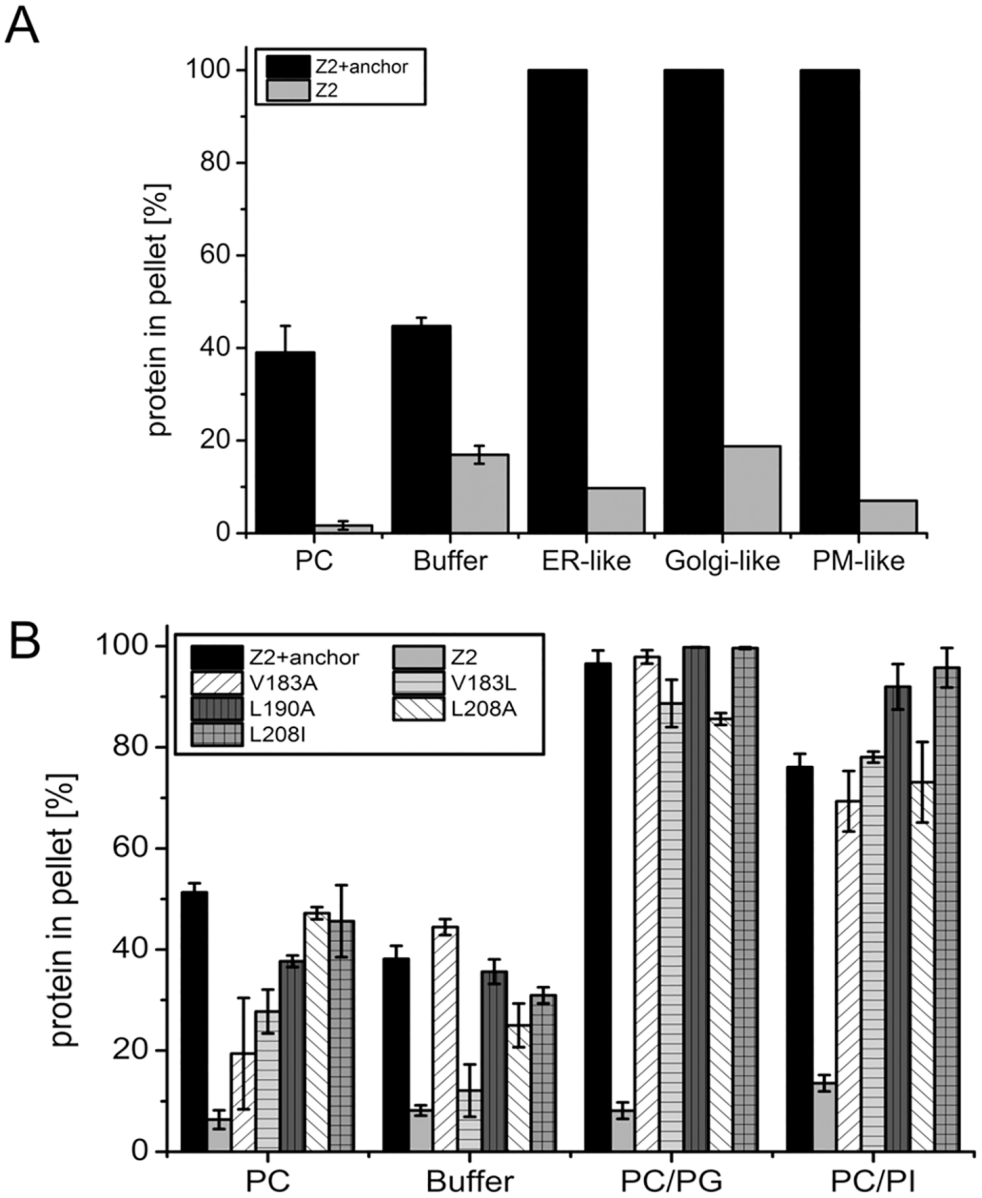
Determinants of intracellular location of E^rns^. In (A), binding of Z2+achor to lipid vesicles with lipid compositions mimicking those of cellular membranes is shown. ER (ER-like), the Golgi apparatus (Golgi-like) or the plasma membrane (PM-like). The percentage of DMPC (PC), DMPE (PE), DMPS (PS), PI (PI), DMPG (PG) und sphingosine (Sph) are given in Table 1. (B) Influence of mutations shown before to influence intracellular retention of E^rns^ [25]. The tested exchanges are indicated in the graph.

The E^rns^ protein is translocated cotranslationally into the ER but is not transported to the plasma membrane with the bulk flow. Earlier work had shown that the membrane anchor region of the protein contains the intracellular localization signal [25]. It has been shown before that the lipid composition of the organelle membrane can influence the localization of proteins. Biological membranes contain a complex mixture of different phospholipids. To find out whether the E^rns^ anchor preferentially binds to vesicles mimicking one of these membranes we mixed the different lipids according to the amounts given in Table 1. In the lipid binding assays these differently composed vesicles showed very similar rates of Z2+anchor binding (Fig 10A). Thus, the lipid binding assay does not provide any clue that the ER localization of E^rns^ is due to a preference for the ER membrane.

**Table 1:** Composition of lipid mixtures mimicking the intracellular membranes.

in %	PC	PE	PS	PI	Sph	PG
**ER-like**	56	22	5	9	6	2
**Golgi-like**	51	18	6	10	14	0
**PM-like**	35	23	14	6	23	0

Composition of the ER (ER-like), the Golgi apparatus (Golgi-like) or the plasma membrane (PM-like). The percentage of DMPC (PC), DMPE (PE), DMPS (PS), PI (PI), DMPG (PG) and sphingosine (Sph) are given.

The characterization of the E^rns^ intracellular localization signal revealed that three amino acids, Val183, Leu190 and Leu208, which are all orientated towards one face of the helix, are of major importance for retention. Mutation of these residues increased E^rns^ secretion and promoted plasma membrane localization of E^rns^/CD72 fusion proteins [25]. However, the function of these residues for retention remained unclear. To look whether exchange of these residues influences lipid binding of the membrane anchor, Z2+anchor constructs with mutations V183A, V183L, L190A, L208A or L208I were established, the respective proteins expressed in bacteria, purified and analyzed in the lipid binding assay. This experiment revealed only small differences with regard to the recovery rate of the different proteins in the vesicle pellet (Fig 10B). Especially, the interaction with the vesicles containing the negatively charged lipids DMPG or DMPI was very similar. These results show that reduced membrane binding of the mutated anchor is not causative for the changes with regard to retention and secretion of the proteins observed in the cell system.

## Discussion

The pestiviral glycosylated surface protein E^rns^ represents a very interesting viral factor with multiple functions for virus replication and interaction with its natural host. On the one hand, E^rns^ is an essential component of the virion responsible for a crucial step during infection of cells [14, 46–48]. On the other hand, E^rns^ exhibits RNase function, a highly unusual feature for a viral structural protein [15, 16]. E^rns^ belongs to the T2 RNases, a widely distributed class of ancient RNases with mostly undefined functions [17–19]. The enzymatic activity of E^rns^ is not essential for virus viability, since inactivation of the RNase by mutation of one of the two His residues in the active center of the protein did not impair virus replication kinetics in tissue culture cells [20–22]. In the natural host, RNase negative viruses showed strong attenuation which resulted in an almost apathogenic phenotype [21, 22]. Nevertheless, infection with RNase negative pestiviruses induces highly protective immunity, so that RNase negative viruses represent promising candidates for novel vaccines against pestiviruses.

As outlined above, the RNase function of E^rns^ represents a virulence factor of pestiviruses. Moreover, animal studies with in utero infections of fetuses revealed that the RNase is important for establishing the very special type of persistent infections of pestiviruses [23]. The mechanisms behind these findings and the RNA target hydrolyzed by the enzyme are still obscure. Several recently published investigations provided evidence for interference of the RNase with the type 1 interferon response of the infected host [23, 24, 31]. Due to its nature as a viral surface protein translocated cotranslationally into the ER lumen, a function in the cytoplasm of the infected cell can be excluded. In fact, *in vitro* analyses showed that the RNase efficiently degrades the viral genome so that direct contact between RNase and viral RNA would interfere with pestivirus propagation [49]. A small proportion of the E^rns^ protein is secreted from the infected cell [26, 28, 29]. Hydrolysis of extracellular RNA, internalization and RNA degradation in endosomal compartments as well as prevention of IFN-1 release by plasmacytoid dendritic cells coming into contact with infected cells were described as functions exhibited by the E^rns^ RNase [24, 26, 27]. A plausible explanation for these observations implies that the secreted E^rns^ is responsible for the virulence factor function of the RNase. Accordingly, we decided to analyze the molecular basis for the equilibrium between secretion and intracellular retention of E^rns^ and we started with work aiming at identification of the E^rns^ membrane anchor. This work was necessary since E^rns^ does not contain a typical membrane anchor but could be shown to be associated to lipid bilayers also in the absence of any other viral protein [29, 33]. We found out that E^rns^ is hooked to membranes via a sequence located at its carboxyterminus. Binding was resistant to high ionic strength and other chemical challenges indicating that the interaction was mainly hydrophobic and much stronger than found for typical peripheral membrane proteins. The E^rns^ membrane anchor can fold into a long amphipathic helix which is responsible for membrane binding [29]. The hydrophobic face of the helix is inserted into the lipid bilayer slightly tilted and in a way that proton exchange with the aqueous phase is prevented for a rather long stretch of amino acids in the middle part of the anchor [32].

The type of membrane anchor identified in E^rns^ was not described before for other surface proteins. For further characterization we tested a large number of different mutations affecting the helical folding of the anchor or the amphipathic character of the helix. Every modification of the E^rns^ anchor resulted in increased secretion of E^rns^ form the cell. Thus, the integrity of the amphipathic helix is important for intracellular retention of E^rns^. We therefore wanted to find out whether such mutations reduce the affinity of the E^rns^ anchor for membranes, which would be an easy explanation for increased secretion when intracellular retention of the protein is dependent on membrane binding, e.g. because interaction of E^rns^ with a membrane-bound factor is essential for retention. Such a model implies that non membrane bound E^rns^ is secreted, so that reducing the affinity of the E^rns^ anchor for lipid membranes would increase the amount of secreted protein. To analyze this point we used the above described *in vitro* sedimentation assay allowing determination of lipid binding in the absence of putatively troublesome other proteins. For different reasons, this assay is not suitable for identification of small changes of protein/lipid binding efficiency. However, it allowed us to test a larger number of test proteins in a reasonable time with a sufficient sensitivity to compare the results with data on protein secretion from infected cells, which is increased by up to a factor of 10 for the mutants tested here. A similar assay system was used before in work on the membrane binding of the *E*.*coli* protein MinD [50], phosphocholine cytidylyltransferase (CCT) [51] or proteins with pleckstrin homology domains [36].

To be able to easily purify the test protein and determine membrane binding of the E^rns^ anchor in the absence of the bulky and highly glycosylated ectodomain of the protein, the anchor sequence was fused to a tag sequence. Due to the fact that the vesicles used for pull down were prepared from synthetic lipids, the assay system allowed to analyze the influence of lipid composition on protein recovery from the pellet. Enhanced specific binding of the E^rns^ anchor was observed for vesicles containing lipids with negative netcharge. This preference for lipids with negatively charged headgroups is a common feature of most proteins anchored via amphipathic helices which can be contributed to the positive netcharge of their membrane binding domains (for review see [43]). In contrast to the published data the E^rns^ anchor exhibits enhanced binding also to vesicles containing negatively charged phosphatidylinositol (PI, see above) and different phosphatidylinositolphosphate variants carrying more than one negative charge. The increase of negative charge resulting from inclusion of these lipids as well as changing the net charge of the E^rns^ anchor sequence itself, however, did not increase Z2+anchor binding showing that charge alone does not dominate binding efficiency (not shown). It has to be kept in mind, that the anchor sequence, despite its positive net charge, contains a considerable number of potentially negatively charged residues, all but one clustered in the aminoterminal half. It has been shown for other amphipathic helices containing Glu or Asp residues that these residues are protonated when the helix is bound to the membrane. This effect was attributed to the presence of anionic lipids and represents a further argument for the preference of these helices for negatively charged membranes (reviewed in [43]). The experiments with Z2+anchor constructs containing mutations in the anchor sequence showed that changes leading in the context of E^rns^ expressed in cells to secretion rates of up to 7–8 times of wt level did not significantly reduce membrane binding in the sedimentation assay. This was true for insertion of single residues at different positions resulting in a twist of one part of the helix with regard to the other and thereby destroying the continuity of the amphipathic character. Similarly, helix breaking mutations like exchange of different amino acids for proline had only limited effects on vesicle binding. Even the combination of both twisting and secondary structure destruction by insertion of single proline residues did not abrogate lipid binding but led to a reduction of less than 50%.

At first glance these results seem surprising. A possible explanation for the robust lipid binding of the E^rns^ anchor is given by its length. Compared to many other amphipathic helices conferring membrane association the E^rns^ anchor with its roughly 60 amino acids is very long. Amphipathic helices of similar length are present in CCT (51 amino acids) and α-Synuclein containing two amphipathic segments of 36 and 37 residues separated by 9 residues [43]. In contrast, the BMV-1a sequence contains only 25 residues and the MinD anchor is composed of only 10 to 19 amino acids five of which are charged [43, 50]. Similarly, amphipathic helices responsible for (temporary) attachment of a variety of viral proteins to intracellular membranes are usually quite short [52–63]. Thus, very few hydrophobic residues adopting the conformation of an amphipathic helix upon membrane contact are sufficient for membrane binding. It therefore seems plausible that partial disruption of the amphipathic helix in the E^rns^ membrane anchor does not prevent lipid binding since sufficiently long properly folded and orientated regions are preserved. This hypothesis is supported by the results of experiments conducted with Z2 tag constructs containing truncated forms or only internal parts of the membrane anchor. All tested parts exhibited at least residual lipid binding. Importantly, these experiments revealed that the carboxyterminal half of the anchor represents the most effective region for lipid binding. This region is colinear with the carboxyterminal half of peptide B and with peptide C that both showed a high degree of helical folding in circular dichroism (CD) spectroscopy in the presence of DMPC/DMPG phospholipids [25]. Moreover, the carboxyterminal sequence, shown here to be of major importance for lipid binding, was analyzed in CD and orientated CD spectroscopy as well as in NMR spectroscopy before (E^rns^ΔN in [32]). These analyses clearly revealed that this fragment of E^rns^ folds into a helix that is inclined into the lipid bilayer. The structure model of the carboxyterminal half of the E^rns^ anchor fits well with the lipid binding data presented here. Taken together it can be concluded that residues ~194 to 227 represent the main determinant for membrane binding. However, the preceding part of the anchor shows also a significant affinity for lipids so that mutations, insertions and limited truncation of the anchor do not abrogate lipid binding.

The latter conclusion was also true for an E^rns^ anchor fusion protein expressed within cells. In earlier studies we showed that a major amount of GFP fused at its carboxyterminus with the C-terminal part of E^rns^ is found in the membrane fraction when it is expressed without signal sequence so that it is not translocated to the ER but stays in the cytoplasm of the cells. It therefore can be concluded that the E^rns^ sequence confers association of the fusion protein to the cytoplasmic side of intracellular membranes. Recovery in the membrane fraction was not only observed for fusions with the complete anchor but also for N- or C-terminally truncated forms [33]. This finding stands in marked contrast to the results conducted with E^rns^ proteins with carboxyterminal truncations or various internal deletions. All these mutated proteins were almost exclusively found in the supernatant. Loss of the five C-terminal residues FGAYA of the anchor was sufficient for nearly complete secretion of the protein [33].

The membrane anchor contains a signal for intracellular localization of the E^rns^ protein. E^rns^ expressed within a cell does not show up on the plasma membrane but accumulates in a not further defined part of the ER [25]. We were able to show that fusion of the region containing the amphipathic helix to proteins like CD72 or CD8α was able to determine intracellular localization of these proteins that without this sequence are transported to the cell surface with the bulk flow. The molecular basis for the specific localization of the protein is not known. It has been shown for some proteins that their retention in certain compartments is influenced by the specific lipid composition of the compartment’s membrane [64–68]. We therefore investigated whether the E^rns^ anchor shows different affinity to different synthetic membranes mimicking the composition of the bilayers surrounding the intracellular compartments. However, the sedimentation assays showed that the anchor binds equivalently well to all tested membranes. Thus, the increased secretion observed for many E^rns^ mutants with alterations in the anchor region is definitely not due to largely decreased affinity to membranes. Accordingly, E^rns^ secretion rates are determined by factors beyond mere lipid binding.

The work on the E^rns^ retention signal has led to identification of a set of three hydrophobic amino acids, all located on one side of the helix, which are especially important for retention. Mutations affecting these residues (Val183, Leu190 and Leu208) resulted in increased secretion of E^rns^, but more importantly, to increased localization of E^rns^ on the cell surface [25]. When testing the equivalent mutations in the BVDV CP7 E^rns^ in the lipid binding assay striking differences in comparison with the wt protein were not observed. This result was somewhat expected and was in agreement with the other mutation experiments showing that the anchor has a robust lipid affinity that cannot easily be disturbed by mutating single amino acids.

The most important result of the analyses described above is the discrepancy between data obtained with the mutations when investigating the retention/secretion of E^rns^ from transfected cells and the lipid binding assay. Mutations leading to severely increased secretion rates had no or only minor influence on binding to lipid vesicles. Major truncations could be tolerated with regard to vesicle binding (this report) as well as attachment of E^rns^ anchor tagged GFP to intracellular membranes [33]. The main difference between the E^rns^ and GFP expression assays is the localization of the proteins in the ER lumen or cytoplasm, respectively. These findings show that the equilibrium between secretion and intracellular retention of E^rns^ must be controlled by other factors than the mere affinity to membranes. Since the variation of the lipid composition in a way that mimics different intracellular membranes also had no striking effect on E^rns^ vesicle binding, it is obvious that variation in affinity to different lipid compositions can hardly be responsible for intracellular location of the protein or the observed defined equilibrium between retention and secretion. Accordingly, one or more other host cellular factors, most likely protein(s), should interact with E^rns^ for its retention. Identification of this or these factor(s) and elucidation of their mode of action is necessary to understand the molecular basis of E^rns^ secretion in detail.

It is important to notice that mutations affecting the membrane anchor of E^rns^ are not causative for E^rns^ secretion in general but result only in increased secretion. Also part of the wt E^rns^ synthesized within a cell is secreted into the cell free supernatant. Secretion can only occur when not only intracellular retention but also membrane binding is abrogated, because otherwise the mutant protein would be found at the cell surface. Thus, it can be concluded, that E^rns^ seems to exist in two forms, an intracellular membrane bound form and a secreted version. It is a common feature of proteins anchored by amphipathic helices that their membrane binding is transient with extrinsic factors regulating lipid association. Examples are the MinD protein that is attached to membranes after binding of ATP and subsequently orchestrates binding of the other Min-system components to the membrane, a process important for regulation of bacterial cell division. CCT interconverts between a soluble inactive form and a lipid-bound active form. This process is regulated by membrane lipid composition and the phosphorylation state of CCT. Upon membrane binding the enzymatic activity of CCT is activated. Also viral proteins were found to be activated only upon association to membranes via amphipathic helices. The methyltransferase activity of the NSP1 protein of Semliki Forest virus is an example which becomes active upon membrane binding [54]. Also the GTPase activity residing in the NS4B protein of human hepatitis C virus is only activated by membrane binding [69]. Further studies will have to concentrate on the question how the equilibrium between secretion and retention of E^rns^ is established in order to understand this feature that is important for the virulence factor activity of this fascinating multifunctional protein.

## Conclusion

Pestivirus persistence requires blocking innate immunity by secretion of a viral RNase usually bound to intracellular membranes or the viral envelope via its amphipathic carboxyterminus. Our lipid binding experiments show that the increased secretion of E^rns^ membrane anchor mutants from cells is not due to decreased affinity of the anchor for membranes. This result contradicts a model according to which the secretion/retention equilibrium of E^rns^ directly correlates with the ratio of membrane bound versus soluble protein. Thus, it is highly likely that cellular non-lipid factors control secretion of this viral immunomodulatory protein.

## References

[1] Thiel HJ, Plagemann PGW, Moennig V. Pestiviruses In: Fields BN, Knipe DM, Howley PM, editors. Fields Virology. 3rd Philadelphia, New York: Lippincott—Raven Publishers; 1996 p. 1059–73.

[2] Simmonds P, Becher P, Collett MS, Gould EA, Heinz FX, Meyers G, et al Flaviviridae In: King AMQ, Lefkowitz E, Adams MJ, Carstens EB, Fauquet CM, editors. Virus Taxonomy Ninth Report of the International Committee on Taxonomy of Viruses. San Diego, USA: Academic Press; 2012 p. 1003–20.

[3] Lindenbach BD, Thiel HJ, Rice CM. Flaviviridae: The Viruses and Their Replication In: Knipe DM, Howley PM, editors. Fields Virology. 5th Philadelphia, New York: Lippincott—Raven Publishers; 2007 p. 1101–52.

[4] Bauhofer O, Summerfield A, Sakoda Y, Tratschin JD, Hofmann MA, Ruggli N. Classical swine fever virus Npro interacts with interferon regulatory factor 3 and induces its proteasomal degradation. JVirol. 2007;81(7):3087–96. 17215286 10.1128/JVI.02032-06 PMC1866024

[5] Chen Z, Rijnbrand R, Jangra RK, Devaraj SG, Qu L, Ma Y, et al Ubiquitination and proteasomal degradation of interferon regulatory factor-3 induced by Npro from a cytopathic bovine viral diarrhea virus. Virology. 2007;366(2):277–92. 17531282 10.1016/j.virol.2007.04.023 PMC2000802

[6] Doceul V, Charleston B, Crooke H, Reid E, Powell PP, Seago J. The Npro product of classical swine fever virus interacts with IκBα, the NF-κB inhibitor. JGenVirol. 2008;89(Pt 8):1881–9. 10.1099/vir.0.83643-0 18632959

[7] Gil LH, Ansari IH, Vassilev V, Liang D, Lai VC, Zhong W, et al The amino-terminal domain of bovine viral diarrhea virus Npro protein is necessary for alpha/beta interferon antagonism. JVirol. 2006;80(2):900–11. 16378992 10.1128/JVI.80.2.900-911.2006 PMC1346884

[8] Hilton L, Moganeradj K, Zhang G, Chen YH, Randall RE, McCauley JW, et al The NPro product of bovine viral diarrhea virus inhibits DNA binding by interferon regulatory factor 3 and targets it for proteasomal degradation. JVirol. 2006;80(23):11723–32. 16971436 10.1128/JVI.01145-06 PMC1642611

[9] La Rocca SA, Herbert RJ, Crooke H, Drew TW, Wileman TE, Powell PP. Loss of interferon regulatory factor 3 in cells infected with classical swine fever virus involves the N-terminal protease, Npro. JVirol. 2005;79(11):7239–47. 15890962 10.1128/JVI.79.11.7239-7247.2005 PMC1112113

[10] Ruggli N, Bird BH, Liu L, Bauhofer O, Tratschin JD, Hofmann MA. N(pro) of classical swine fever virus is an antagonist of double-stranded RNA-mediated apoptosis and IFN-alpha/beta induction. Virology. 2005;340(2):265–76. 16043207 10.1016/j.virol.2005.06.033

[11] Ruggli N, Summerfield A, Fiebach AR, Guzylack-Piriou L, Bauhofer O, Lamm CG, et al Classical swine fever virus can remain virulent after specific elimination of the interferon regulatory factor 3-degrading function of Npro. JVirol. 2009;83(2):817–29. 18987150 10.1128/JVI.01509-08 PMC2612357

[12] Ruggli N, Tratschin JD, Schweizer M, McCullough KC, Hofmann MA, Summerfield A. Classical swine fever virus interferes with cellular antiviral defense: evidence for a novel function of N(pro). JVirol. 2003;77(13):7645–54. 12805464 10.1128/JVI.77.13.7645-7654.2003 PMC164809

[13] Seago J, Hilton L, Reid E, Doceul V, Jeyatheesan J, Moganeradj K, et al The Npro product of classical swine fever virus and bovine viral diarrhea virus uses a conserved mechanism to target interferon regulatory factor-3. JGenVirol. 2007;88(Pt 11):3002–6. 10.1099/vir.0.82934-0 17947522

[14] Thiel HJ, Stark R, Weiland E, Rümenapf T, Meyers G. Hog cholera virus: molecular composition of virions from a pestivirus. J Virol. 1991;65:4705–12. 1870198 10.1128/jvi.65.9.4705-4712.1991 PMC248926

[15] Hulst MM, Himes G, Newbigin E, Moormann RJM. Glycoprotein E2 of classical swine fever virus: expression in insect cells and identification as a ribonuclease. Virology. 1994;200:558–65. 8178442 10.1006/viro.1994.1218

[16] Schneider R, Unger G, Stark R, Schneider-Scherzer E, Thiel HJ. Identification of a structural glycoprotein of an RNA virus as a ribonuclease. Science. 1993;261:1169–71. 8356450 10.1126/science.8356450

[17] Deshpande RA, Shankar V. Ribonucleases from T2 family. Critical reviews in microbiology. 2002;28(2):79–122. . 12109772 10.1080/1040-840291046704

[18] Irie M, Ohgi K. Ribonuclease T2. Methods in enzymology. 2001;341:42–55. . 11582795 10.1016/s0076-6879(01)41144-x

[19] Krey T, Bontems F, Vonrhein C, Vaney MC, Bricogne G, Rümenapf T, et al Crystal structure of the pestivirus envelope glycoprotein E(rns) and mechanistic analysis of its ribonuclease activity. Structure. 2012;20(5):862–73. 10.1016/j.str.2012.03.018 . 22579253

[20] Hulst MM, Panoto FE, Hoekman A, van Gennip HG, Moormann RJ. Inactivation of the RNase activity of glycoprotein E rns of classical swine fever virus results in a cytopathogenic virus. J Virol. 1998;72:151–7. 9420210 10.1128/jvi.72.1.151-157.1998 PMC109359

[21] Meyer C, Von Freyburg M, Elbers K, Meyers G. Recovery of virulent and RNase-negative attenuated type 2 bovine viral diarrhea viruses from infectious cDNA clones. 2002;76(16):8494–503. 12134054 10.1128/JVI.76.16.8494-8503.2002 PMC155120

[22] Meyers G, Saalmüller A, Büttner M. Mutations abrogating the RNase activity in glycoprotein e(rns) of the pestivirus classical swine fever virus lead to virus attenuation. J Virol. 1999;73(12):10224–35. 10559339 10.1128/jvi.73.12.10224-10235.1999 PMC113076

[23] Meyers G, Ege A, Fetzer C, von FM, Elbers K, Carr V, et al Bovine viral diarrhoea virus: Prevention of persistent foetal infection by a combination of two mutations affecting the Erns RNase and the Npro protease. JVirol. 2007;0(JVI):02372–06. 10.1128/JVI.02372-06 PMC1866084 17215285

[24] Python S, Gerber M, Suter R, Ruggli N, Summerfield A. Efficient sensing of infected cells in absence of virus particles by plasmacytoid dendritic cells is blocked by the viral ribonuclease E(rns.). PLoS pathogens. 2013;9(6):e1003412 10.1371/journal.ppat.1003412 23785283 PMC3681750

[25] Burrack S, Aberle D, Bürck J, Ulrich AS, Meyers G. A new type of intracellular retention signal identified in a pestivirus structural glycoprotein. FASEB journal: official publication of the Federation of American Societies for Experimental Biology. 2012;26(8):3292–305. Epub 2012/05/03. 10.1096/fj.12-207191 . 22549508

[26] Magkouras I, Mätzener P, Rümenapf T, Peterhans E, Schweizer M. RNase-dependent inhibition of extracellular, but not intracellular, dsRNA-induced interferon synthesis by Erns of pestiviruses. JGenVirol. 2008;89(Pt 10):2501–6. 10.1099/vir.0.2008/003749-0 18796719

[27] Mätzener P, Magkouras I, Rümenapf T, Peterhans E, Schweizer M. The viral RNase E(rns) prevents IFN type-I triggering by pestiviral single- and double-stranded RNAs. Virus Res. 2009;140(1–2):15–23. 10.1016/j.virusres.2008.10.015 19041350

[28] Rümenapf T, Unger G, Strauss JH, Thiel HJ. Processing of the envelope glycoproteins of pestiviruses. J Virol. 1993;67:3288–95. 8388499 10.1128/jvi.67.6.3288-3294.1993 PMC237670

[29] Tews BA, Meyers G. The Pestivirus Glycoprotein Erns Is Anchored in Plane in the Membrane via an Amphipathic Helix. JBiolChem. 2007;282(45):32730–41. 10.1074/jbc.M706803200 17848558

[30] Iqbal M, Poole E, Goodbourn S, McCauley JW. Role for bovine viral diarrhea virus Erns glycoprotein in the control of activation of beta interferon by double-stranded RNA. JVirol. 2004;78(1):136–45. 14671095 10.1128/JVI.78.1.136-145.2004 PMC303375

[31] Zürcher C, Sauter KS, Mathys V, Wyss F, Schweizer M. Prolonged activity of the pestiviral RNase Erns as an interferon antagonist after uptake by clathrin-mediated endocytosis. J Virol. 2014;88(13):7235–43. 10.1128/JVI.00672-14 24741078 PMC4054414

[32] Aberle D, Muhle-Goll C, Bürck J, Wolf M, Reisser S, Luy B, et al Structure of the membrane anchor of pestivirus glycoprotein E(rns), a long tilted amphipathic helix. PLoS pathogens. 2014;10(2):e1003973 10.1371/journal.ppat.1003973 24586172 PMC3937272

[33] Fetzer C, Tews BA, Meyers G. The carboxy-terminal sequence of the pestivirus glycoprotein E(rns) represents an unusual type of membrane anchor. JVirol. 2005;79(18):11901–13. 16140766 10.1128/JVI.79.18.11901-11913.2005 PMC1212594

[34] Wyatt LS, Moss B, Rozenblatt S. Replication-deficient vaccinia virus encoding bacteriophage T7 RNA polymerase for transient gene expression in mammalian cells. Virology. 1995;210(1):202–5. 7793072 10.1006/viro.1995.1332

[35] Schägger H, Jagow Gv. Tricine- sodium dodecyl sulfate-polyacrylamide gel electrophoresis for the separation of proteins in the range from 1 to 100 kDa. Anal Biochem. 1987;166:368–79. 2449095 10.1016/0003-2697(87)90587-2

[36] Harlan JE, Hajduk PJ, Yoon HS, Fesik SW. Pleckstrin homology domains bind to phosphatidylinositol-4,5-bisphosphate. Nature. 1994;371(6493):168–70. 10.1038/371168a0 . 8072546

[37] Avanti Polar Lipids Inc http://www.avantilipids.com/index.php?option=com_content&view=article&id=1384&Itemid=372

[38] Morissey JH. Morrissey Lab Protocol for Preparing Phospholipid Vesicles (SUV) by Sonication. http://www.avantilipids.com/images/PDF/MorrisseyLabProtocolForPrepSuvBySonication.pdf

[39] Tews BA, Schürmann EM, Meyers G. Mutation of cysteine 171 of pestivirus E rns RNase prevents homodimer formation and leads to attenuation of classical swine fever virus. JVirol. 2009;83(10):4823–34. 19264773 10.1128/JVI.01710-08 PMC2682062

[40] Bogomolovas J, Simon B, Sattler M, Stier G. Screening of fusion partners for high yield expression and purification of bioactive viscotoxins. Protein expression and purification. 2009;64(1):16–23. 10.1016/j.pep.2008.10.003 . 18983922

[41] Tautz N, Meyers G, Stark R, Dubovi EJ, Thiel HJ. Cytopathogenicity of a pestivirus correlated with a 27 nucleotide insertion. J Virol. 1996;70(11):7851–8. 8892907 10.1128/jvi.70.11.7851-7858.1996 PMC190856

[42] Huth JR, Bewley CA, Jackson BM, Hinnebusch AG, Clore GM, Gronenborn AM. Design of an expression system for detecting folded protein domains and mapping macromolecular interactions by NMR. Protein science: a publication of the Protein Society. 1997;6(11):2359–64. 10.1002/pro.5560061109 9385638 PMC2143577

[43] Cornell RB, Taneva SG. Amphipathic helices as mediators of the membrane interaction of amphitropic proteins, and as modulators of bilayer physical properties. Current protein & peptide science. 2006;7(6):539–52. . 17168787 10.2174/138920306779025675

[44] Szeto TH, Rowland SL, Habrukowich CL, King GF. The MinD membrane targeting sequence is a transplantable lipid-binding helix. J Biol Chem. 2003;278(41):40050–6. 10.1074/jbc.M306876200 . 12882967

[45] Liu L, Westler WM, den Boon JA, Wang X, Diaz A, Steinberg HA, et al An amphipathic alpha-helix controls multiple roles of brome mosaic virus protein 1a in RNA replication complex assembly and function. PLoS pathogens. 2009;5(3):e1000351 10.1371/journal.ppat.1000351 19325881 PMC2654722

[46] Hulst MM, Moormann RJ. Inhibition of pestivirus infection in cell culture by envelope proteins E(rns) and E2 of classical swine fever virus: E(rns) and E2 interact with different receptors. JGenVirol. 1997;78 (Pt 11):2779–87. 10.1099/0022-1317-78-11-2779 9367363

[47] Hulst MM, Moormann RJ. Erns protein of pestiviruses. Methods Enzymol. 2001;342:431–40. 11586914 10.1016/s0076-6879(01)42564-x

[48] Weiland E, Ahl R, Stark R, Weiland F, Thiel HJ. A second envelope glycoprotein mediates neutralization of a pestivirus, hog cholera virus. J Virol. 1992;66(6):3677–82. 1583727 10.1128/jvi.66.6.3677-3682.1992 PMC241151

[49] Windisch JM, Schneider R, Stark R, Weiland E, Meyers G, Thiel HJ. RNase of Classical swine fever virus: Biochemical characterization and inhibition by virus-neutralizing monoclonal antibodies. J Virol. 1996;70:352–8. 8523547 10.1128/jvi.70.1.352-358.1996 PMC189824

[50] Zhou H, Lutkenhaus J. Membrane binding by MinD involves insertion of hydrophobic residues within the C-terminal amphipathic helix into the bilayer. Journal of bacteriology. 2003;185(15):4326–35. 12867440 10.1128/JB.185.15.4326-4335.2003 PMC165746

[51] Arnold RS, DePaoli-Roach AA, Cornell RB. Binding of CTP:phosphocholine cytidylyltransferase to lipid vesicles: diacylglycerol and enzyme dephosphorylation increase the affinity for negatively charged membranes. Biochemistry. 1997;36(20):6149–56. 10.1021/bi970023z . 9166786

[52] Moradpour D, Brass V, Penin F. Function follows form: the structure of the N-terminal domain of HCV NS5A. Hepatology. 2005;42(3):732–5. Epub 2005/08/24. 10.1002/hep.20851 . 16116650

[53] Moradpour D, Gosert R, Egger D, Penin F, Blum HE, Bienz K. Membrane association of hepatitis C virus nonstructural proteins and identification of the membrane alteration that harbors the viral replication complex. Antiviral research. 2003;60(2):103–9. Epub 2003/11/26. . 14638405 10.1016/j.antiviral.2003.08.017

[54] Spuul P, Salonen A, Merits A, Jokitalo E, Kääriäinen L, Ahola T. Role of the amphipathic peptide of Semliki forest virus replicase protein nsP1 in membrane association and virus replication. J Virol. 2007;81(2):872–83. 10.1128/JVI.01785-06 17093195 PMC1797454

[55] Brass V, Pal Z, Sapay N, Deleage G, Blum HE, Penin F, et al Conserved determinants for membrane association of nonstructural protein 5A from hepatitis C virus and related viruses. J Virol. 2007;81(6):2745–57. Epub 2006/12/29. 10.1128/JVI.01279-06 17192310 PMC1866014

[56] Dubuisson J, Penin F, Moradpour D. Interaction of hepatitis C virus proteins with host cell membranes and lipids. Trends in cell biology. 2002;12(11):517–23. Epub 2002/11/26. . 12446113 10.1016/s0962-8924(02)02383-8

[57] Gouttenoire J, Castet V, Montserret R, Arora N, Raussens V, Ruysschaert JM, et al Identification of a novel determinant for membrane association in hepatitis C virus nonstructural protein 4B. J Virol. 2009;83(12):6257–68. Epub 2009/04/10. 10.1128/JVI.02663-08 19357161 PMC2687391

[58] Gouttenoire J, Montserret R, Kennel A, Penin F, Moradpour D. An amphipathic alpha-helix at the C terminus of hepatitis C virus nonstructural protein 4B mediates membrane association. J Virol. 2009;83(21):11378–84. Epub 2009/08/21. 10.1128/JVI.01122-09 19692468 PMC2772773

[59] Gouttenoire J, Moradpour D, Penin F. Surprises from the crystal structure of the hepatitis C virus NS2-3 protease. Hepatology. 2006;44(6):1690–3. Epub 2006/11/30. 10.1002/hep.21449 . 17133477

[60] Gouttenoire J, Penin F, Moradpour D. Hepatitis C virus nonstructural protein 4B: a journey into unexplored territory. Reviews in medical virology. 2010;20(2):117–29. Epub 2010/01/14. 10.1002/rmv.640 . 20069613

[61] Gouttenoire J, Roingeard P, Penin F, Moradpour D. Amphipathic alpha-helix AH2 is a major determinant for the oligomerization of hepatitis C virus nonstructural protein 4B. J Virol. 2010;84(24):12529–37. Epub 2010/10/12. 10.1128/JVI.01798-10 20926561 PMC3004355

[62] Schmidt-Mende J, Bieck E, Hugle T, Penin F, Rice CM, Blum HE, et al Determinants for membrane association of the hepatitis C virus RNA-dependent RNA polymerase. J Biol Chem. 2001;276(47):44052–63. Epub 2001/09/15. 10.1074/jbc.M103358200 . 11557752

[63] Lampio A, Kilpeläinen I, Pesonen S, Karhi K, Auvinen P, Somerharju P, et al Membrane binding mechanism of an RNA virus-capping enzyme. J Biol Chem. 2000;275(48):37853–9. 10.1074/jbc.M004865200 . 10984480

[64] Bretscher MS, Munro S. Cholesterol and the Golgi apparatus. Science. 1993;261(5126):1280–1. . 8362242 10.1126/science.8362242

[65] Pelham HR, Munro S. Sorting of membrane proteins in the secretory pathway. Cell. 1993;75(4):603–5. . 8242736 10.1016/0092-8674(93)90479-A PMC7133381

[66] Simons K, Sampaio JL. Membrane organization and lipid rafts. Cold Spring Harbor perspectives in biology. 2011;3(10):a004697 10.1101/cshperspect.a004697 21628426 PMC3179338

[67] Mouritsen OG. Model answers to lipid membrane questions. Cold Spring Harbor perspectives in biology. 2011;3(9):a004622 10.1101/cshperspect.a004622 21610116 PMC3181035

[68] Tian A, Baumgart T. Sorting of lipids and proteins in membrane curvature gradients. Biophysical journal. 2009;96(7):2676–88. 10.1016/j.bpj.2008.11.067 19348750 PMC2711293

[69] Einav S, Elazar M, Danieli T, Glenn JS. A nucleotide binding motif in hepatitis C virus (HCV) NS4B mediates HCV RNA replication. J Virol. 2004;78(20):11288–95. 10.1128/JVI.78.20.11288-11295.2004 15452248 PMC521822

